# Abi1 loss drives prostate tumorigenesis through activation of EMT and non-canonical WNT signaling

**DOI:** 10.1186/s12964-019-0410-y

**Published:** 2019-09-18

**Authors:** Disharee Nath, Xiang Li, Claudia Mondragon, Dawn Post, Ming Chen, Julie R. White, Anita Hryniewicz-Jankowska, Tiffany Caza, Vladimir A. Kuznetsov, Heidi Hehnly, Tamara Jamaspishvili, David M. Berman, Fan Zhang, Sonia H. Y. Kung, Ladan Fazli, Martin E. Gleave, Gennady Bratslavsky, Pier Paolo Pandolfi, Leszek Kotula

**Affiliations:** 1 Department of Urology, Upstate Cancer Center, SUNY Upstate Medical University, 750 East Adams Street, Syracuse, New York 13210 USA; 2 Department of Biochemistry and Molecular Biology, SUNY Upstate Medical University, Syracuse, NY 13210 USA; 3 Cancer Research Institute, Beth Israel Deaconess Cancer Center, Department of Medicine and Pathology, Beth Israel Deaconess Medical Center, Harvard Medical School, Boston, MA 02215 USA; 4 Laboratory of Comparative Pathology, Memorial Sloan-Kettering Cancer Center, New York, NY 10065 USA; 5 Department of Pathology and Medicine, SUNY Upstate Medical University, Syracuse, NY 13210 USA; 6 Department of Cell and Developmental Biology, SUNY Upstate Medical University, Syracuse, NY 13210 USA; 7 Department of Pathology and Molecular Medicine and Division of Cancer Biology & Genetics, Queen’s Cancer Research Institute, Queen’s University, 10 Stuart St, Kingston, ON K7L 3N6 Canada; 8 Department of Urologic Sciences, Vancouver Prostate Centre, University of British Columbia, Vancouver, BC V6H 3Z6 Canada; 9 Bioinformatics Institute, A-STAR, Singapore, 138671 Singapore; 10 Present address: Department of Pathology, Duke University School of Medicine, Durham, NC 27710 USA; 11 Duke Cancer Institute, Duke University, Durham, NC 27710 USA; 12 Department of Cytobiochemistry, Faculty of Biotechnology, University of Wroclaw, ul. F. Joliot-Curie 14a, 50-383 Wroclaw, Poland

## Abstract

**Background:**

Prostate cancer development involves various mechanisms, which are poorly understood but pointing to epithelial mesenchymal transition (EMT) as the key mechanism in progression to metastatic disease. *ABI1,* a member of WAVE complex and actin cytoskeleton regulator and adaptor protein, acts as tumor suppressor in prostate cancer but the role of *ABI1* in EMT is not clear.

**Methods:**

To investigate the molecular mechanism by which loss of *ABI1* contributes to tumor progression, we disrupted the *ABI1* gene in the benign prostate epithelial RWPE-1 cell line and determined its phenotype. Levels of ABI1 expression in prostate organoid tumor cell lines was evaluated by Western blotting and RNA sequencing. ABI1 expression and its association with prostate tumor grade was evaluated in a TMA cohort of 505 patients and metastatic cell lines.

**Results:**

Low ABI1 expression is associated with biochemical recurrence, metastasis and death (*p* = 0.038). Moreover, ABI1 expression was significantly decreased in Gleason pattern 5 vs. pattern 4 (*p* = 0.0025) and 3 (*p* = 0.0012), indicating an association between low ABI1 expression and highly invasive prostate tumors. Disruption of *ABI1* gene in RWPE-1 cell line resulted in gain of an invasive phenotype, which was characterized by a loss of cell-cell adhesion markers and increased migratory ability of RWPE-1 spheroids. Through RNA sequencing and protein expression analysis, we discovered that *ABI1* loss leads to activation of non-canonical WNT signaling and EMT pathways, which are rescued by re-expression of ABI1. Furthermore, an increase in STAT3 phosphorylation upon *ABI1* inactivation and the evidence of a high-affinity interaction between the FYN SH2 domain and ABI1 pY421 support a model in which ABI1 acts as a gatekeeper of non-canonical WNT-EMT pathway activation downstream of the FZD2 receptor.

**Conclusions:**

ABI1 controls prostate tumor progression and epithelial plasticity through regulation of EMT-WNT pathway. Here we discovered that ABI1 inhibits EMT through suppressing FYN-STAT3 activation downstream from non-canonical WNT signaling thus providing a novel mechanism of prostate tumor suppression.

**Electronic supplementary material:**

The online version of this article (10.1186/s12964-019-0410-y) contains supplementary material, which is available to authorized users.

## Background

Prostate cancer (PCa) is the most prevalent visceral cancer affecting the male population worldwide and the second highest cause of cancer-related death in the Western population [1]. While indolent PCa is treatable, metastatic cancer invariably has a high mortality rate [2], thus warranting studies of PCa progression mechanisms and development of new therapeutic targets.

Several growth control pathways have been implicated in high-risk prostate cancer. Prostate tissue growth is driven by androgen. Prostate tumors exhibit different levels of androgen dependency and are characterized by deregulation of androgen pathway signaling [3]. Aggressive prostate cancer has been linked to Androgen Receptor (AR) activation, which occurs through multiple mechanisms, including intra-tumor androgen production [4]. AR pathway upregulation coincides with activation of PI3-kinase [5] or RAS/MAPK kinase, indicating the importance of targeting these pathways in high-risk tumors [6]. Bioinformatic inquiries over the last decade have resulted in a better understanding of signaling pathways and genetic alterations associated with prostate cancer initiation [7] and metastatic progression [8, 9]. Our previous studies implicated *ABI1* downregulation in high-risk prostate tumors [10], but the mechanism underlying Abi1 tumor suppressor activity is unclear.

Epithelial to mesenchymal transition (EMT) is a key pathway in prostate tumor progression. EMT is characterized by the loss of cell-cell adhesion markers, such E-cadherin and β-catenin, accompanied by an increase in cell migration and invasiveness due to activation of a specific transcriptional program. Transcriptional regulators, such as TWIST, SNAI1, SNAI2, ZEB1, and ZEB2, repress E-cadherin expression, while others promote the expression of mesenchymal differentiation markers, such as N- and/or R-cadherin and vimentin, as well as the expression of cellular matrix and focal adhesion proteins that promote motility, such as focal adhesion kinase and integrins [11]. EMT is also accompanied by increased activity of matrix metalloproteinases, which leads to degradation of the extracellular matrix and increases the ability of cells to invade and metastasize. When the invading cells seed the metastasic site, they may undergo the reverse process, mesenchymal to epithelial transition (MET), and adapt to the local microenvironment [12]. EMT has been linked to advanced prostate cancer, enhanced metastatic potential, neuroendocrine transdifferentiation, and castrate resistance prostate cancer (CRPC) [13]. There is increasing evidence that androgen deprivation therapy (ADT) therapy itself, in the long run, might induce EMT and treatment resistance [14, 15], and hence, it has become an important focus of research investigations.

There is strong evidence that EMT can be activated in prostate cancer not only by growth factor receptors and TGFβ [16] or by specific pathway mutations [8] but also by activation of the WNT pathway [8, 17]. In the canonical WNT pathway, β-catenin translocates from the plasma membrane to the nucleus and activates a transcriptional program that promotes EMT [18]. In other cancers, such as colon cancer, the canonical WNT pathway is typically associated with APC mutations or deletions [19], but canonical WNT pathway alterations are also seen in advanced prostate cancer, which together with β-catenin and RNF43 mutations occur in approximately 15% of cases [8]. In a recent study, the noncanonical β-catenin-independent WNT pathway, represented by WNT5, FZD2, FYN, and STAT3 [20], was found to be associated with high-risk prostate cancer [21].

STAT3 is a master regulator of tumor progression and EMT. STAT3 plays a pivotal role in progression of many tumor types, including prostate cancer tumors [22] [23]. STAT3 is activated downstream from a variety of cellular receptors, including growth factor receptors, (EGF, VEGF, PDGF), non-receptor tyrosine kinases (such as ABL, SFK), G-protein-coupled receptors, Toll-like receptors, and cytokine receptors (such as IL6 receptor) [24]. In addition to its tumor-autonomous role, STAT3 helps to suppress tumor immune responses through its activation in myeloid-derived suppressor cells [25]. In high-grade tumors, STAT3 activation (STAT-pY705) is associated with increased levels of Hsp27 [23]. However, how STAT3 activation is regulated to promote progression of prostate tumors is still unclear.

The hallmark mechanism of EMT in tumor progression is loss of cell-cell adhesion [11, 26]. Epithelial cell-to-cell adhesion is regulated at zonula adherens, where the transmembrane protein E-cadherin acts through α- and β-catenin to link adherens junctions to the actin cytoskeleton. The heteropentameric WAVE complex comprising ABI 1/2/3, WAVE 1/2/3, CYFIP 1/2, NAP 1/2, and BRK1 regulates ARP2/3-dependent actin dynamics at cell junctions [27]. ABI1, a component of the WAVE complex, was proposed to act upstream of mDia in cell-cell junctions in siRNA studies [28]. Genetic loss of *Abi1* causes destabilization and degradation of the WAVE complex [29], consistent with in vitro studies [30, 31]. Upregulation of WAVE3 has been associated with EMT in tumor progression [32]. However, the role of ABI1 in promoting EMT is not known.

ABI1 regulates tumor invasiveness through integrin signaling. Loss of *Abi1* phenocopies integrin α4 knockout during embryonic development in the mouse [33]. Integrin recycling occurs through macropinocytosis via rapid recycling of dorsal ruffles, and ABI1 is the critical regulator of dorsal ruffling [29] and fluid phase macropinocytosis [34]. Abi1 was linked to regulation of integrin in preclinical models of breast cancer [35], and leukemia [36]. However, the impact of *ABI1* loss on integrin expression in the prostate epithelium or in prostate tumors is not known.

*ABI1* is associated with 10p deletions in prostate cancer [37], along with *ABI1* mutations and deletions being identified within the gene itself [10, 38]. In addition, the prostate cancer cell line LNCaP has a mutation in *ABI1* [37]; re-expression of ABI1 inhibits prostate tumor xenograft growth [38]. Disruption of *Abi1* expression in the mouse leads to prostatic intraepithelial neoplasia (PIN) with high penetrance in all lobes, and loss of Abi1 is associated with downregulation of E-cadherin, which together with the PIN phenotype suggests a role of Abi1 in prostate tumor initiation [38]. Moreover, ABI1 is downregulated in castration-resistant prostate cancer, thus indicating that it might be important in tumor progression [10].

Here, we investigated the mechanistic roles for ABI1 loss in prostate cancer progression. Analysis of metastatic prostate tumor cell lines and a prostate tumor tissue microarray indicated that low Abi1 expression preferentially affects high-grade prostate tumors. Modeling of *ABI1* loss via CRISPR-mediated KO in the non-tumorigenic prostate cell line RWPE-1 defined the expression signature, which was consistent with activation of EMT through increased levels of pY705 STAT3. Our mechanistic analysis supports the hypothesis that Abi1 acts downstream of the non-canonical WNT receptor FZD2 and upstream of the active FYN-STAT3 axis to control epithelial plasticity through an EMT program.

## Results

### Low Abi1 expression is associated with high-grade prostate cancer and biochemical recurrence

**Fig. 1:**
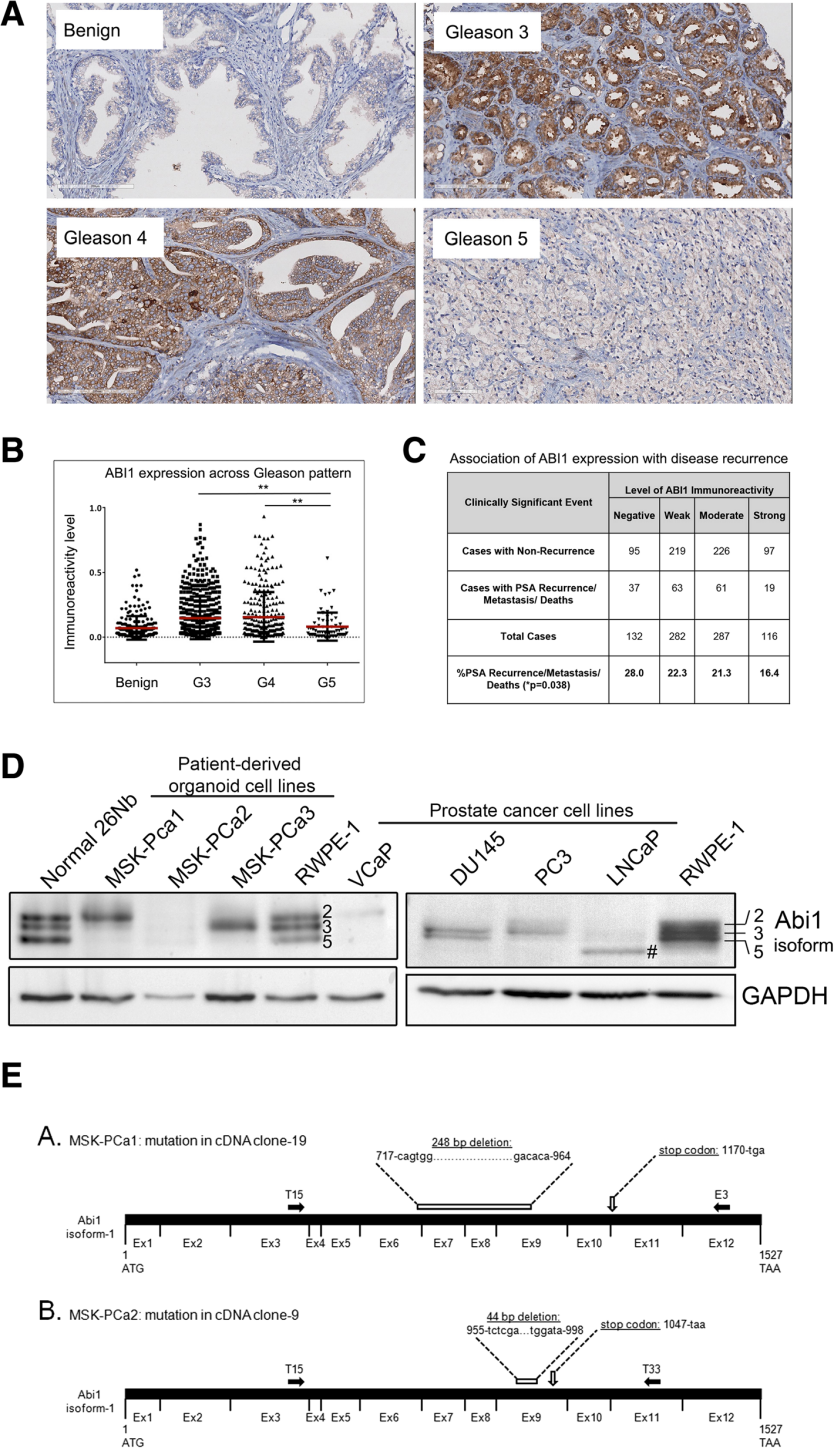
ABI1 expression is downregulated during prostate tumor progression. Prostate cancer tissue microarray (*n* = 505 patients) was stained for ABI1 to evaluate its expression during prostate tumor progression. (**a**) Representative ABI1 expression staining in Gleason 3, 4, and 5 patterns, as indicated; *Benign* indicates normal prostate epithelium. (**b**) Comparison of ABI1 expression among Gleason patterns, 3, 4 and 5 indicating significant downregulation of ABI1 in Gleason 5 vs. 3, (*p* = 0.0012); or 4 (*p* = 0.0025) (one-way ANOVA, multiple comparison). (**c**) Analysis of ABI1 expression in the tissue microarray indicating an association between low ABI1 expression and clinically significant events, such as biochemical recurrence, a metastatic event, and death; *p* = 0.038; 99% confidence interval: 0.03308–0.4292; Linear-by-Linear association exact test (Sytel Studio-9). (**d**) Representative Western blots showing reduced levels of ABI1 in different patient-derived organoid cell lines (left) and immortalized prostate cancer cell lines (right) compared with non-tumor 26Nb and RWPE-1 cells. Numbers next to bands in the *RWPE-1* lane and on the right side of the panel indicate ABI1 isoform designation: top band, isoform 2; middle band, isoform 3; and bottom band, isoform 5 [34, 40]; #, indicates the mutant ABI1 lacking exon 6 in LNCaP [37]. GAPDH was used as the loading control. (**e**) Identification of mutations in the cDNA of ABI1 from organoid cultures of human metastatic prostate cancer cell lines. (A. and B.) Schematic of full-length *ABI1* cDNA (isoform-1) with coding regions of consecutive exons (Ex1-Ex12) indicated (not drawn to scale). The ATG start codon (corresponding to nucleotide 1), TAA stop codon (corresponding to nucleotide 1527), and the T15, E3, and T33 primer sites (filled-in black arrows) are labeled. The T15, E3, and T33 primers [40] were used to selectively amplify *ABI1* cDNA via PCR from the MSK-PCa1 and MSK-PCa2 organoid cell lines. PCR products were subcloned into a pCR-Blunt II TOPO vector and sequenced. A. Schematic of mutation identified in cDNA clone-19 sequence from MSK-PCa1 cells. Sequencing of cDNA clone-19 revealed a 248-bp deletion spanning nucleotides 717 to 964 (from the 3′ end of Exon6, at the junction between Exon 6 and Exon 7, to the middle of Exon 9) that is predicted to result in a frame shift and subsequent stop codon at nucleotide 1170. B. Schematic of mutation identified in cDNA clone-9 from MSK-PCa2 cells. Sequencing of cDNA clone-9 revealed a 44-bp deletion spanning nucleotides 955 to 998 in the middle of Exon 9 that is predicted to result in a frame shift and subsequent stop codon at nucleotide 1047

Our previous studies identified the loss of heterozygosity in *ABI1* gene locus [37], however, the mechanism of ABI1 role as a tumor suppressor in prostate cancer is poorly understood. Staining of a test tumor tissue microarray (32 cases) with ABI1 antibody demonstrated a remarkable loss of ABI1 expression in tumors with high Gleason grade (Additional file 1: Figure S1A). To validate this observation, we analyzed ABI1 expression in large cohort of prostate cancer patients (*n* = 505) (Fig. 1B). The quantification took into consideration intra-core heterogeneity (Additional file 1: Figure S1B), as we scored ABI1 expression in Gleason patterns 3, 4 and 5 separately. Digital scoring results demonstrated significant loss of ABI1 expression in Gleason 5 vs. Gleason 3 (*p* = 0.0012) or Gleason 4 (*p* = 0.0025) patterns, respectively (Fig. 1B). Hence, the most aggressive prostate tumors are associated with low ABI1 expression. To determine clinical significance of this finding we performed correlation analysis of ABI1 expression levels, as determined by digital score, with patient disease recurrence status (PSA biochemical recurrence, metastasis, and death). Low ABI1 expression showed a positive correlation with biochemical recurrence, a metastatic event, and death (*p* = 0.038) across all 505 VPC patients (Fig. 1C) (Contingency Table, Additional file 1: Figure S1B).

### Patient-derived prostate cancer organoid cell lines have low and dysregulated expression of Abi1 isoforms

Here, we analyzed ABI1 expression in prostate tumor cell lines and patient-derived prostate tumor organoid cell lines (PCa1, 2 and 3) [39]. All three PCa organoid cell lines showed reduced levels and loss of one or more ABI1-immunoreactive bands (Fig. 1D, left panel). Low levels of ABI1 expression were also observed in VCaP, (Fig. 1D, left panel), PC3, DU145, and LNCaP prostate cancer cell lines (Fig. 1D, right panel). RWPE-1 (a human non-tumor prostate cell line) expressed relatively high levels of ABI1; RNA sequencing analysis was consistent with RWPE-1 expressing three ABI1 isoforms, isoforms 2, 3, and 5 [40]. ABI1 expression in the normal human prostate organoid cell line (26Nb) matched closely RWPE-1 (Fig. 1D, right panel).

Sequence analysis revealed mutations in ABI1 cDNA in two metastatic PCa cell lines, MSK-PCa1, and MSK-PCa2. In both cases ABI1 cDNA contains large deletions and premature stop codons (Fig. 1E). These mutations may be a cause of lower level of ABI1 and dysregulation of ABI1 isoforms by Western Blotting analysis (Fig. 1D).

### CRISPR/Cas9-mediated ABI1 KO in RWPE-1 cell lines disrupts the WAVE complex

**Fig. 2:**
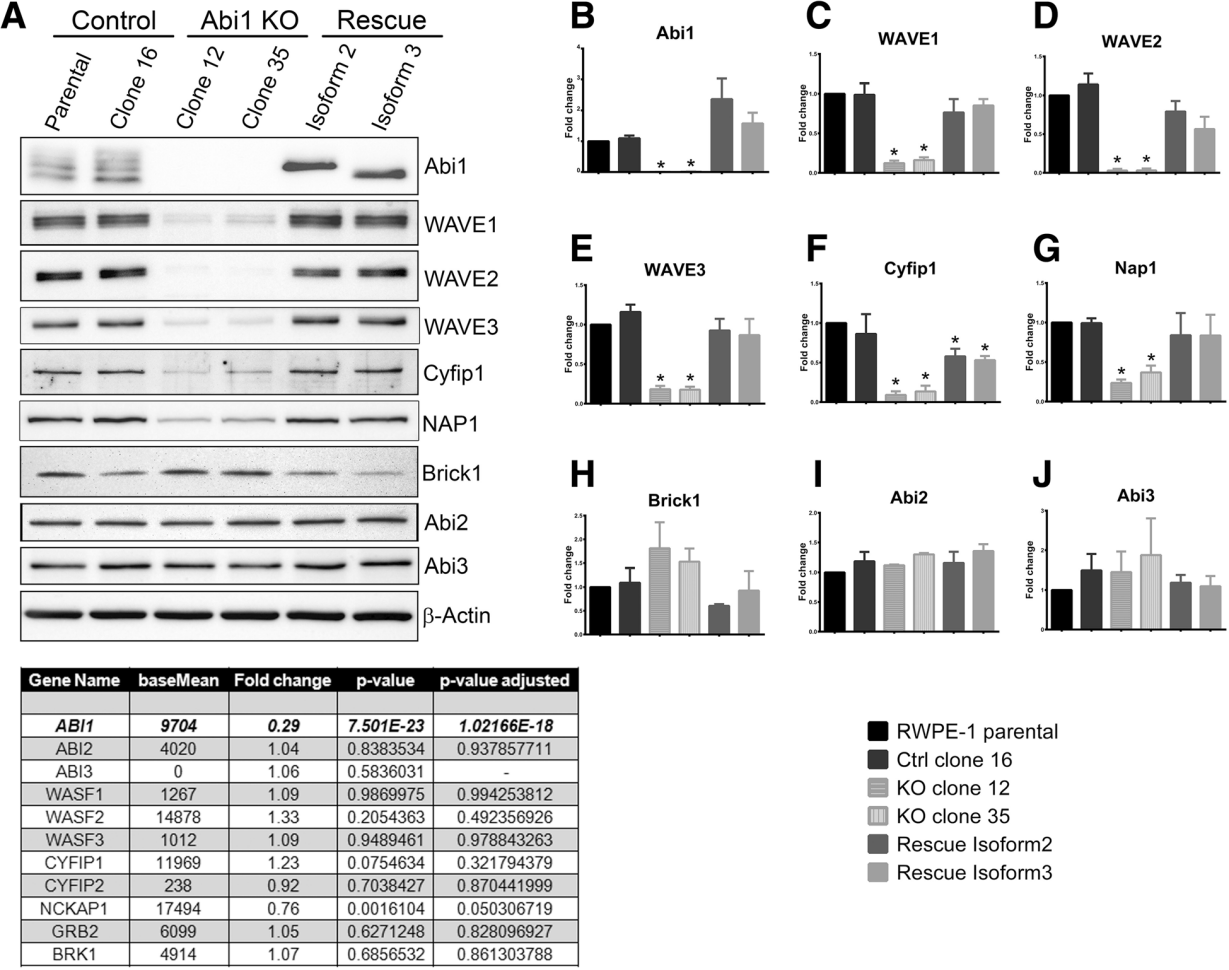
ABI1 KO RWPE-1 cells exhibit reduced WAVE complex levels, which are rescued upon ABI1 re-expression. (**a**) Representative western blots showing reduced levels of different WAVE complex members in the absence of ABI1 in RWPE-1 cells; β-actin was used as the loading control. (**c**) The mRNA levels of all the WAVE complex genes (except ABI1) remained unchanged. The western blots from 3 independent experiments were quantified via densitometry analysis (**b**-**j**). The graphs were generated in Prism, and the data are represented as the mean ± SEM, unpaired Student’s t-test (*p* < 0.05)

To better understand the role of ABI1 as a tumor suppressor in prostate cancer and define ABI1-regulated pathways, we sought to generate a prostate epithelial cell line lacking *ABI1* gene expression. Examining the effects of *ABI1* disruption without the confounding influence of tumorigenic mutations would be very informative; therefore, we selected the benign RWPE-1 prostate epithelial cell line. To generate an ABI1 KO cell line, we used CRISPR-Cas9 technology to obtain genetic knockout in RWPE-1 cells (Material and methods). Following single clone selection and isolation, clones lacking ABI1 expression were confirmed by DNA sequencing to have two alleles of the *ABI1* gene disrupted (Additional file 2: Figure S2). While the RWPE-1 naïve cell line exhibits three prominent expression bands immunoreactive to ABI1 antibody that correspond to Abi1 isoforms 2,3 and 5 [40], no bands were detected in the ABI1 KO clones (Fig. 2A, B). The screening of the clones included examination of WAVE2 expression, which is the key component of the WAVE complex, which is downregulated in mice with genetic knockout of Abi1 [29]. WAVE2 downregulation coincided with loss of Abi1 expression in all KO clones (Additional file 2: Figure S2). As controls, we used clones with an intact Abi1 gene but that underwent all the selection processes (CRISPR-failed clones); these clones also served as controls for off-target effects in the rescue experiments. The rescue experiments were performed with stable clones derived from RWPE-1 ABI1 KO clone 35 expressing isoforms 2 and 3 of ABI1 [34].

First, we determined the levels of the WAVE complex in RWPE cell lines lacking ABI1. ABI1 KO cell lines displayed significant downregulation of the WAVE complex proteins WAVE1, WAVE2, WAVE3, CYFIP1 and NAP1, which was rescued by re-expression of ABI1 (Fig. 2). BRK1 demonstrated modest elevation to no change in expression in Abi1 KO cell lines. Notably, ABI2 and ABI3 were not affected in the Abi1 KO cell lines. The uniform effect of ABI1 disruption on WAVE proteins indicates global downregulation of ABI1-related WAVE complex components in RWPE-1 KO cell lines. Interestingly, this change was only at the protein level; RNA sequencing analysis showed no effect on mRNA levels of the WAVE complex genes (Fig. 2A, bottom panel).

### *ABI1* knockout leads to disrupted cell-cell adhesion via altered levels and localization of the adherens junction proteins E cadherin and β-catenin

**Fig. 3:**
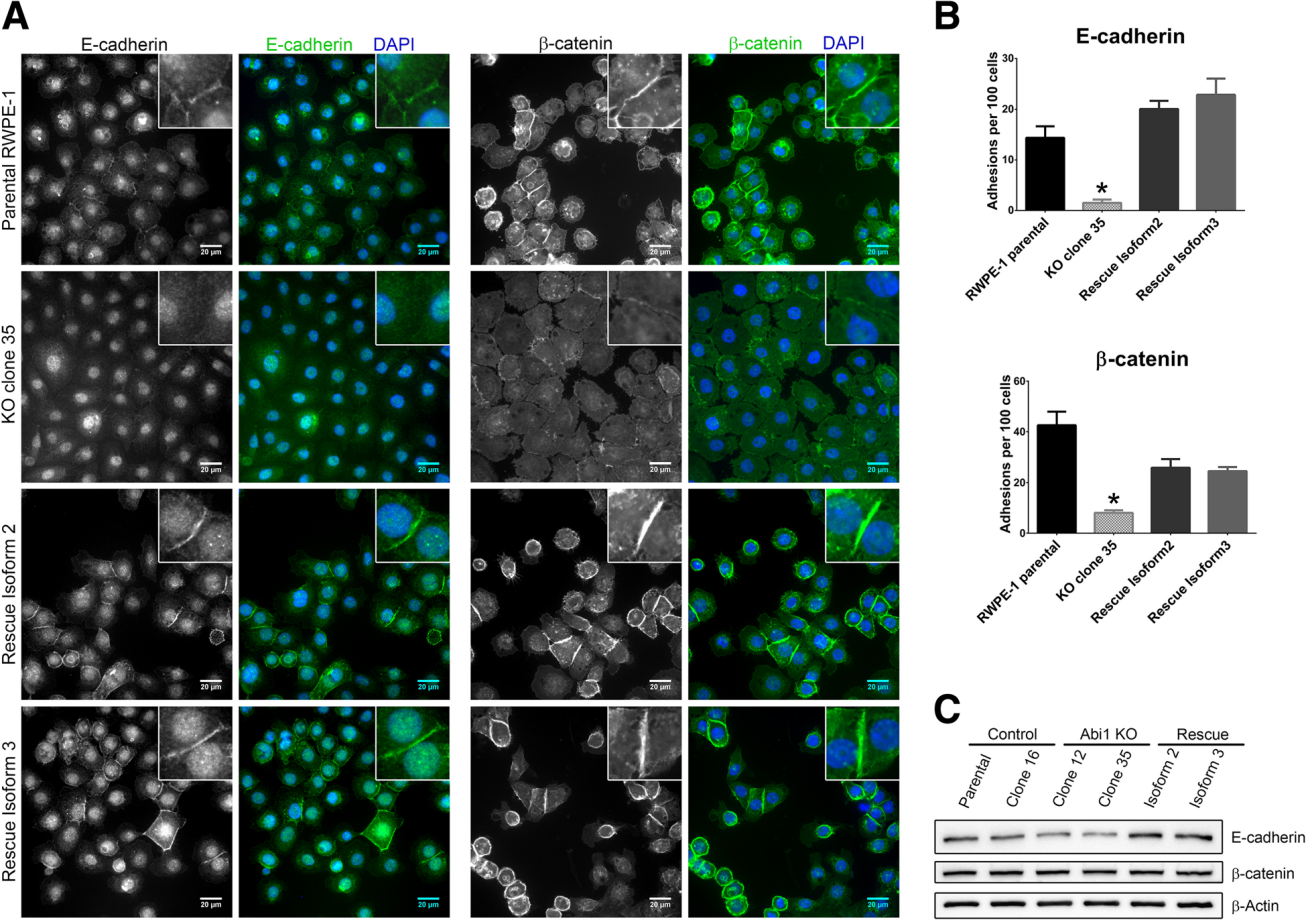
Loss of membrane localization of adherens junction proteins in ABI1 KO cells. (**a**) Immunostaining images showing localization of the adherens junction proteins (left) E-cadherin and (right) β-catenin. (**b**) Quantification of the number of cells with positive junctional staining per 100 cells is shown. Quantification was performed in 6 independent fields per cell line. The graph was generated with Prism software, and the results are represented as the mean ± SEM, unpaired Student’s t-test (*p* < 0.05). (**c**) Western blot analysis of the same proteins showed a modest decrease or no change upon loss of Abi1

Proper cell-cell adhesion is the key to maintaining epithelial integrity, and alterations in cell-cell adhesion have been shown to be key mediators of neoplastic changes. The WAVE complex and ABI1 have been linked to regulation of cell-cell adhesion [27, 28]. Our previous research showed reduced levels of E-cadherin and β-catenin in Abi1 KO mouse prostate tissue [38], and analogous regulation of N-cadherin and β-catenin was demonstrated in MEF cell lines (Additional file 3: Figure S3). Therefore, we looked at levels and localization of adherens junction markers in RWPE-1 ABI1-KO cell lines. Immunostaining data showed a significant reduction in the membrane localization of both proteins. The rescue clones with either ABI1 isoform 2 or 3 restored the levels and localization of E-cadherin and localization of β-catenin (Fig. 3A, B). However, we observed only modest E-cadherin downregulation but unchanged β-catenin expression at the protein level (Fig. 3C).

### Loss of ABI1 leads to increased 2D motility in RWPE-1 Abi1 KO cells

**Fig. 4:**
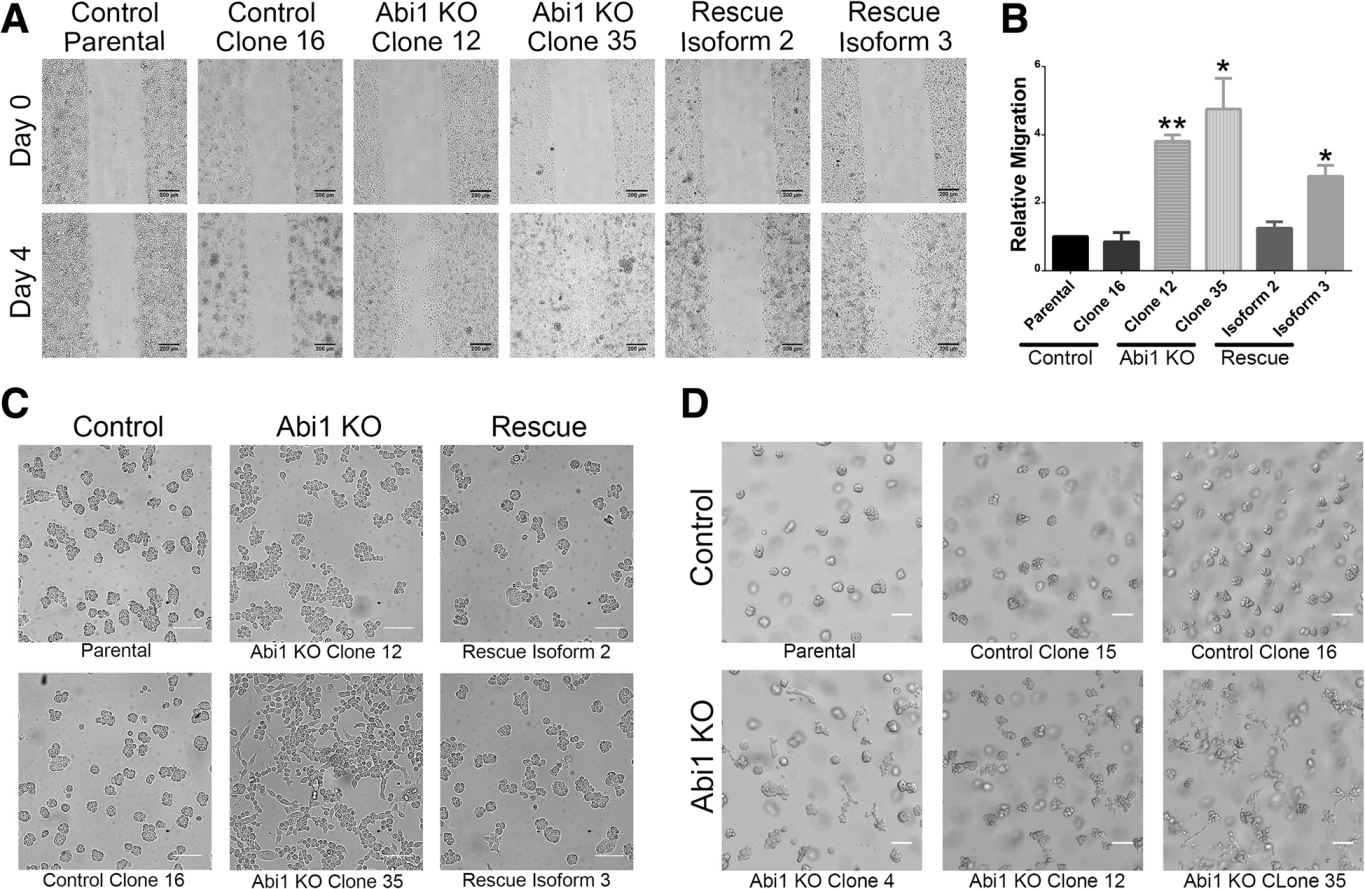
ABI1 KO cells have increased migratory ability in 2D and 3D cultures. (**a**) Representative images from wound healing (scratch-wound) assays at 0 days and 4 days post-scratch demonstrating increased migration of the ABI1 KO cells. Scale bars represent 200 μm. (**b**) The covered area in images was quantified using ImageJ. The graph represents data from *n* = 3 experiments (with 3 independent fields per cell line per experiment) normalized to the parental RWPE-1 cell data. The graph was generated with Prism software, and the results are represented as the mean ± SEM, unpaired Student’s t-test; * (*p* < 0.05); ** (*p* < 0.01). (**c**) Representative images demonstrating altered growth characteristics evidenced by the appearance of the cells in a Matrigel overlay assay. Cells were plated on a chamber slide and overlaid with Matrigel the next day. Images were taken 4 days post-overlay. ABI1 KO cells grew in a 2D culture-like manner (second column), while control RWPE-1 (first column) and ABI1 overexpression cells (third column) primarily grew into spheroids. (**d**) When plated in regular 3D cultures, the ABI1 KO organoids showed invasive/migratory abilities. The images show parental cells, two failed CRISPR control clones (top row) and three different ABI1 KO clones (bottom row) at 4 days post-culture. Scale bars represent 100 μm

ABI1 and the WAVE complex regulates the actin cytoskeleton, and thus, we hypothesized that loss of ABI1 would affect cell adhesion and migration. We investigated the 2D motility of ABI1 KO cells by performing a wound healing assay. Interestingly, the cells lacking Abi1 exhibited an increased migratory ability vs. the parental cell line (Fig. 4A, B). The effect could not be attributed to increased proliferation of ABI1 KO cells because no significant differences in proliferation among the clones were observed, despite activation of phospho-Akt (Additional file 4: Figure S4). Furthermore, the phenotype was almost completely reversed in the Isoform 2-expressing rescue cell line but partially reversed in the Isoform 3-expressing cell line. Hence, ABI1 may have isoform-specific roles in cells [34], and the different isoforms may contribute differently to tumorigenic changes as previously suggested [38].

### ABI1-null cells exhibit a migratory and invasive phenotype in 3D cultures

3D matrices, such as Matrigel, provide a more physiological-like environment for epithelial cells. To delineate the ABI1 phenotype, we plated cells on collagen-coated glass and overlaid cells with Matrigel. Interestingly, the ABI1 null cells grew in a flat 2D-like manner on the surface, while the control cells grew into spheroids upward into the overlaid Matrigel (Fig. 4C). These phenotypes support the notion that Abi1 loss promotes invasive behavior of epithelial cells.

**Fig. 5:**
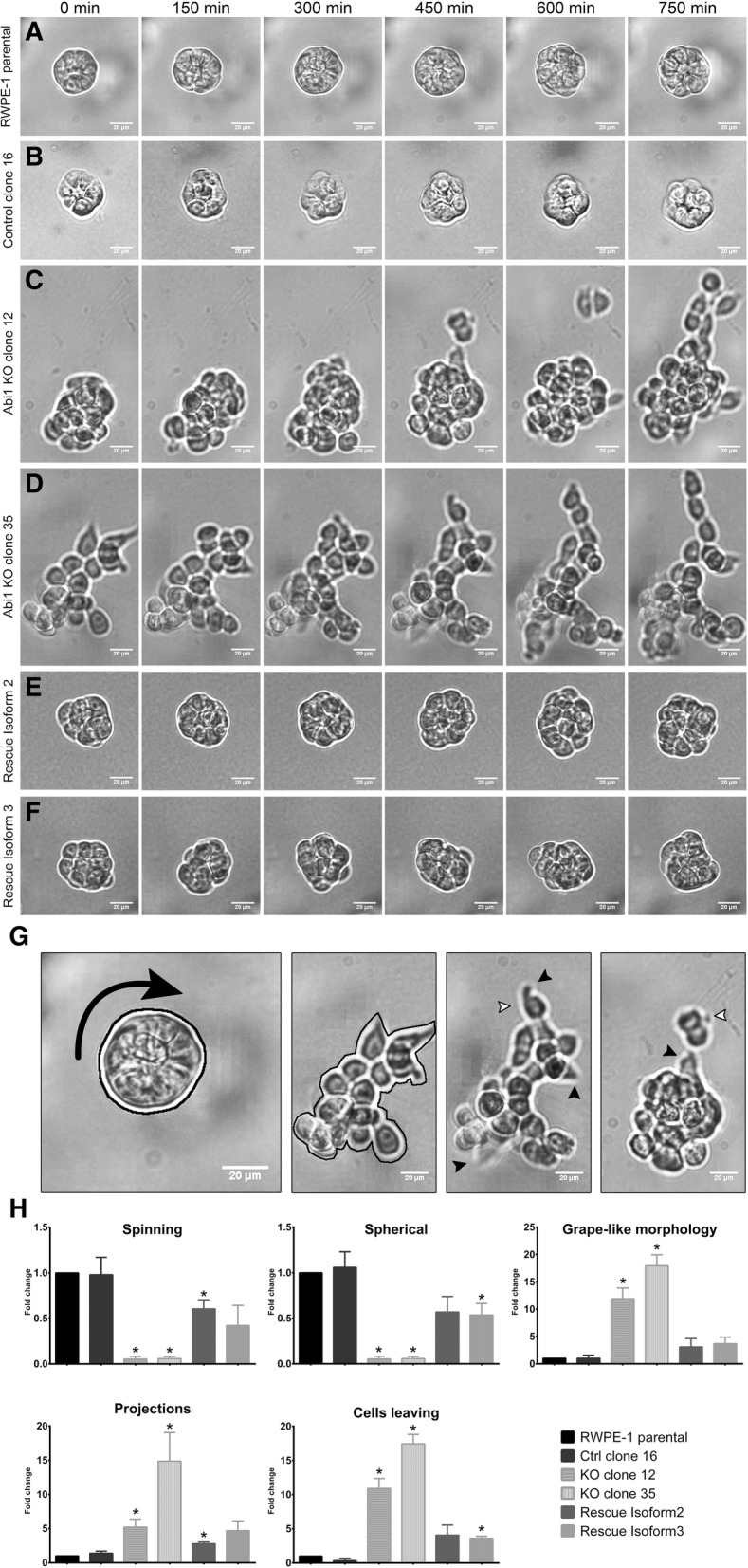
RWPE-1 ABI1 KO spheroids exhibit invasive migratory phenotype in 3D Matrigel cultures. Time-lapse images acquired overnight of representative spheroids showing key features of the spheroids. Cells were plated in 50% Matrigel and allowed to grow for 6 days before live overnight imaging (every 5 min for 14 h). (**a**) RWPE-1 parental, (**b**) Control clone 16, (**c**) ABI1 KO clone 12, (**d**) ABI1 KO clone 35, (**e**) ABI1 rescue Isoform 2, (**f**) ABI1 rescue Isoform 3 cells. (**g**-**h**) Analysis of the morphology and behavior of the spheroids was performed by quantifying the percentage of organoids exhibiting the following characteristics: spinning, spherical shape, grape-like morphology, projections and cell budding/migration from the spheroid. The control spheroids show a spherical shape, while the KO spheroids do not (black outline). KO spheroids feature projections (black filled arrows) and cells trying to escape from the organoid (white arrows). The data represent n = 3 experiments (18–25 spheroids per cell line per experiment) and were normalized to the parental RWPE-1 cell data. The graph was generated with Prism software, and the results are presented as the mean ± SEM, unpaired Student’s t-test with *p*-values * (*p* < 0.05), ** (*p* < 0.01), *** (*p* < .001), and **** (*p* < 0.0001)

Furthermore, when plated and embedded within Matrigel, RWPE-1 parental cells grew into tight clusters or spheroids, with intimately defined cell-cell boundaries; the spheroids constantly spun around their own axes (Fig. 4D top panel, Fig. 5A-B, Additional file 8: Movie S1a, S1b). To investigate if loss of ABI1 altered this organoid morphology and behavior of RWPE-1 cells, we imaged the 3D cultures of both control and ABI1 KO cells live overnight. The ABI1-null spheroids exhibited striking differences compared with control spheroid with intact ABI1 (Fig. 4D, Fig. 5A-B, C-D, Additional file 8: Movie S1a, S1b, S1c, S1d). We observed that most ABI1 KO spheroids lost the tight spherical shape, demonstrated enhanced cell boundaries, and grew more as a loose bunch of cells with a “grape-like morphology” (Fig. 5G). Moreover, a significant fraction of organoids did not show the spinning phenotype (Additional file 8: Movie S1c, S1d). Most interestingly, the cells were found to be sending out lamellipodia-like or filopodia-like projections and were starting to migrate out of the organoid (Fig. 5H). The ABI1-null clones rescued with either isoform 2 or 3 largely regained the naïve cell phenotype (Fig. 5E-F, Additional file 8: Movie S1e, S1f).

### ABI1 depletion promotes upregulation of the EMT pathway

To better understand the molecular changes underlying the ABI1-null phenotype, we performed RNA seq. (Materials and Methods). The wild-type RWPE-1, two CRISPR-control (failed CRISPR) clones, and three ABI1 KO clones were subsequently sequenced, and analysis of RNA sequences indicated no significant differences within the clones with intact ABI1 or between the three ABI1 KO clones. However, 441 differentially expressed genes (DEGs) were identified between the two groups (Additional file 7).

We performed several bioinformatic analyses to define ABI1 functional associations with other pathways based on its gene signature. Pathway analysis (GO, Panther) demonstrated involvement of ABI1 in several developmental processes (Table 1). Reactome analysis indicated association of Abi1 function with cell-surface interaction at the vascular wall and integrin cell-surface pathways (Table 1). Notably, analysis of WAVE complex gene mRNA levels indicated no significant changes in several integral WAVE complex proteins in the absence of ABI1 (Fig. 2A). These results indicate high discordance between the protein and mRNA expression of WAVE complex components and confirm its regulation based on complex stability [30, 31] and the requirement for ABI1 in the complex [29] (Fig. 2).

**Table 1:**
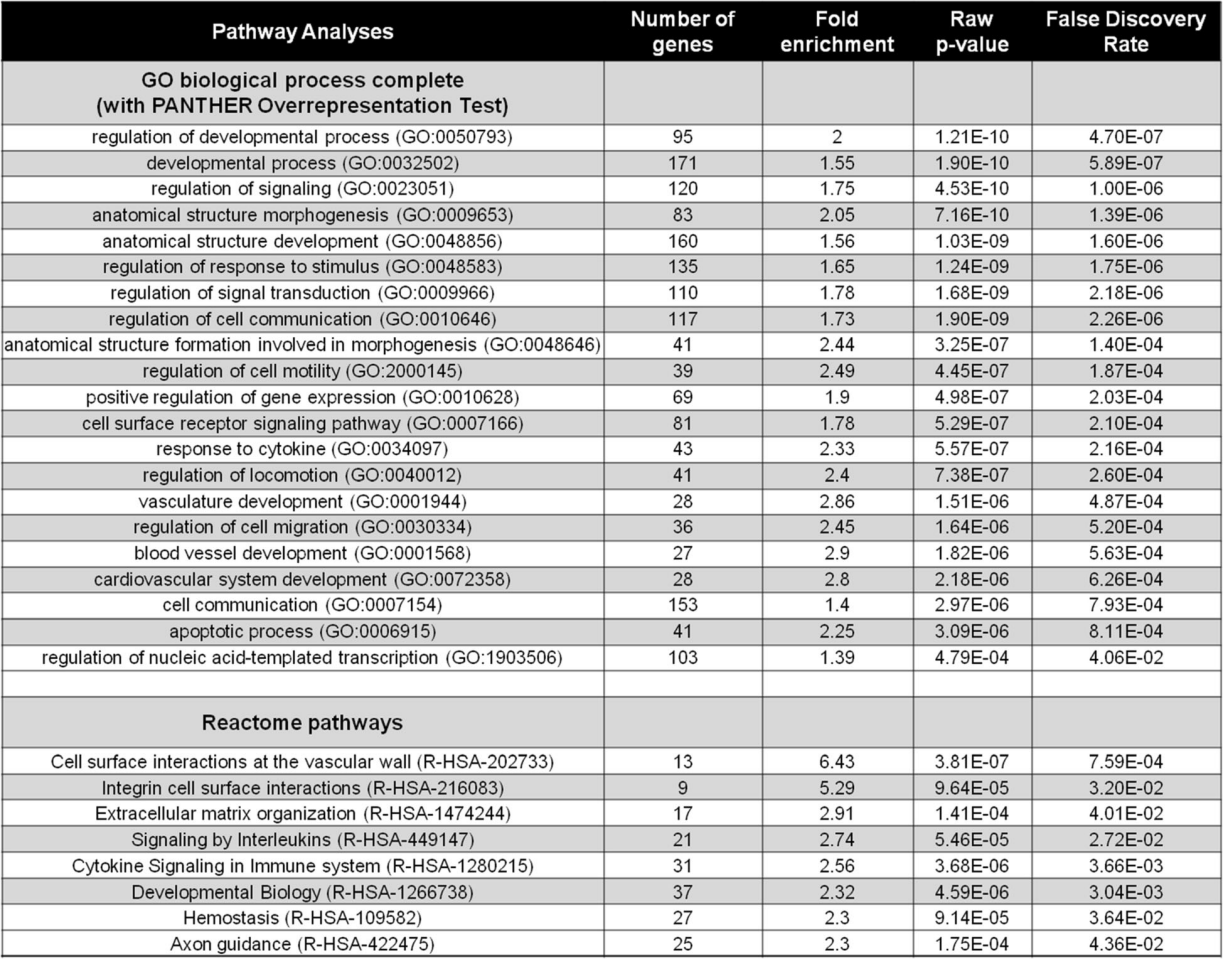
RNA sequencing data analysis

Pathway analysis for *ABI1* gene function based on RNA sequencing data of RWPE ABI1 KO cell lines. Top, GO biological process analysis. Bottom, Reactome pathways. See Additional file 7 for the list 441 *ABI1* differentially expressed genes

**Fig. 6:**
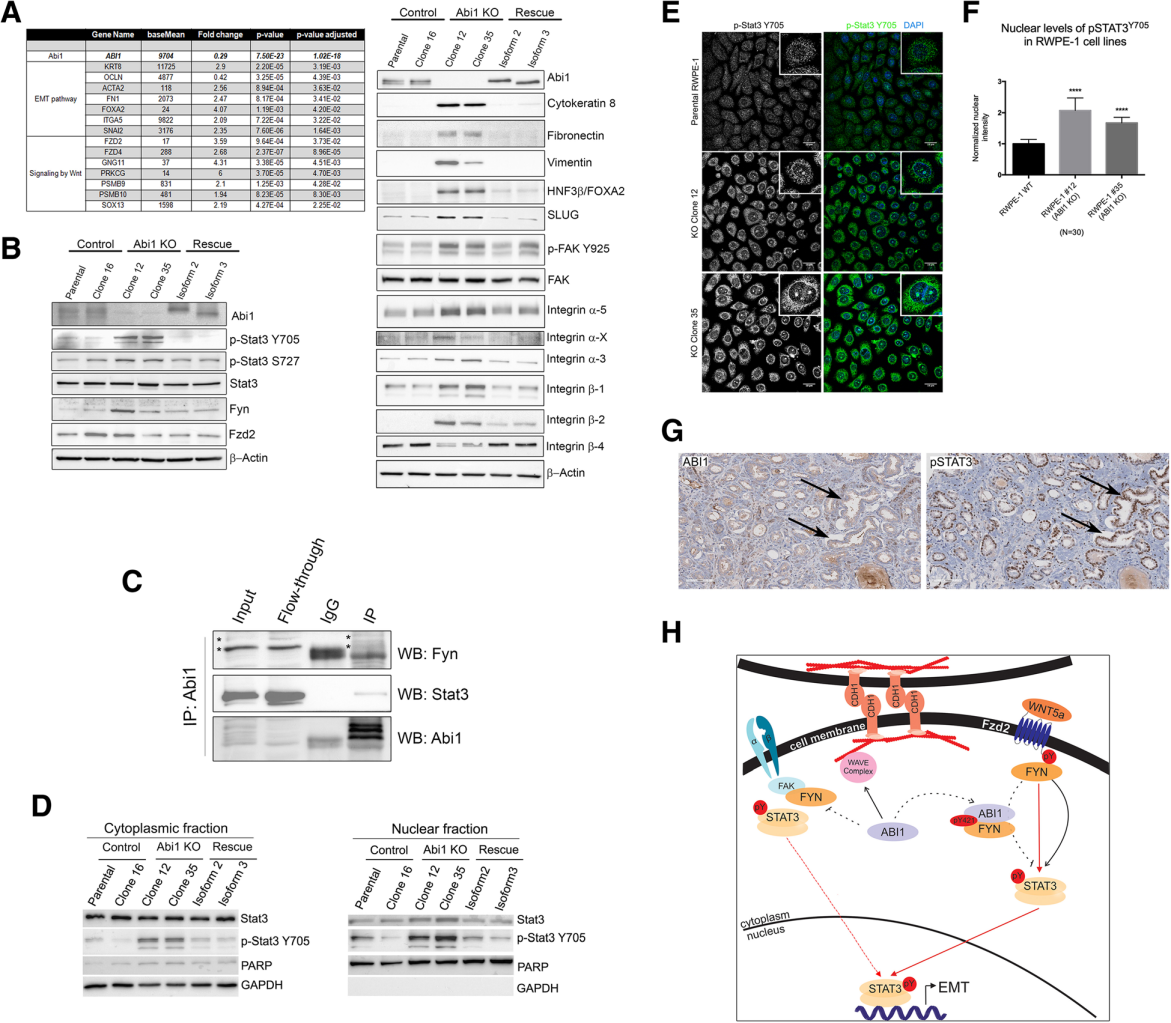
Differential gene expression pattern is correlated with EMT pathways and WNT signaling. The DEGs determined by RNA sequencing demonstrated (**a**) upregulation of EMT pathways and Wnt signaling. Individual genes, fold changes and p-values are listed. Right panel shows representative western blots of several EMT markers and different integrin proteins validate the RNA sequencing findings. (**b**) Western blots showing upregulation of STAT3 phosphorylation (Y705 and S727) and FYN activation. (**c**) ABI1 interacts with STAT3 and FYN in RWPE-1 cells. Western blotting results indicating that FYN (top panel) and STAT3 (middle panel) co-immunoprecipitated with Abi1. Input, RWPE-1 cell lysate; Flow-Through, unbound fraction of the lysate; IgG, control immunoprecipitation lacking anti-Abi1 antibody; IP, immunoprecipitation including the anti-Abi1 antibody. Asterisk in top panel indicates the corresponding bands in fractions. (**d**-**f**) *ABI1* loss promotes nuclear localization of activated STAT3 as determined by pY705 antibody. (**d**) Western blotting analysis of p-STAT3 Y705 expression in cytoplasmic (left panel) or, nuclear fraction (right panel) of RWPE ABI1 KO, control and ABI1-rescued clones. (**e**) Immunofluorescence analysis of p-STAT3 Y705 levels in RWPE ABI1 KO clones. (**f**) Quantification of nuclear p-STAT3 Y705 levels, n = 3, *p* < 0.001. (**g**) An example of inverse correlation of ABI1 and pSTAT3 Y705 expression levels in a prostate tumor. Serial tumor sections were immunostained with antibodies to either ABI1 (left panel), or pSTAT3 Y705 (right panel). Arrows depict two example areas of correlation of low ABI1 and high pSTAT. (**h**) Schematic depicting the proposed mechanism of how Abi1 loss promotes the invasive potential of prostate epithelial cells. Center, ABI1 (*ABI1*) is critical for cell junction maintenance through WAVE complex-mediated actin polymerization; disruption of ABI1 leads to loss of WAVE complex (WAVE complex) stability, resulting in lower levels of E-cadherin (CDH1) at cell-cell contacts and downregulation of cell-cell adhesion. Right, ABI1 pY421 binds with high affinity to FYN-SH2 domain. Disruption of *ABI1* leads to constitutive activation of the SRC family kinase FYN (FYN) to stimulate STAT3 activation and promote its nuclear localization. Nuclear STAT3 promotes transcription of the epithelial-to-mesenchymal transition (EMT) gene program, including extracellular matrix metalloproteinases that promote migration through matrix degradation in the absence of proper cell-cell adhesion. Left, In the absence of ABI1, activated FYN acts on FAK kinase (FAK) to promote integrin signaling, which also supports cell migratory activity. Activated FAK may also directly bind and activate pSTAT3, thus providing another possibility for EMT pathway activation

To understand the mechanism by which ABI1 removal results in gain of an invasive phenotype in RWPE-1 cells, we investigated changes in RNA levels of known tumor-associated pathway genes. Notably, we identified the epithelial-mesenchymal transition (EMT) pathway to be significantly activated, including upregulation of R-cadherin and MMP1 and downregulation of Occludin (Fig. 6A) (Table 2, Additional file 7). Protein levels corroborated the mRNA results for Cytokeratin 8, Fibronectin 1, FOXA2, SLUG, and Integrin α5 (Fig. 6A). There were also changes in several other integrin expression patterns. We observed upregulation of Integrin α3, αX, β1 and β2 (Fig. 6A) and reduced levels of integrin β4. Abi1 re-expression rescued the upregulation of EMT pathway genes as well as the alterations in integrin expression (Fig. 6A). Moreover, upregulation of phosphorylation of FAK at Y925 was evident and was rescued by re-expression of ABI1 Isoform 2 but not isoform 3 (Fig. 6A).

**Table 2:**
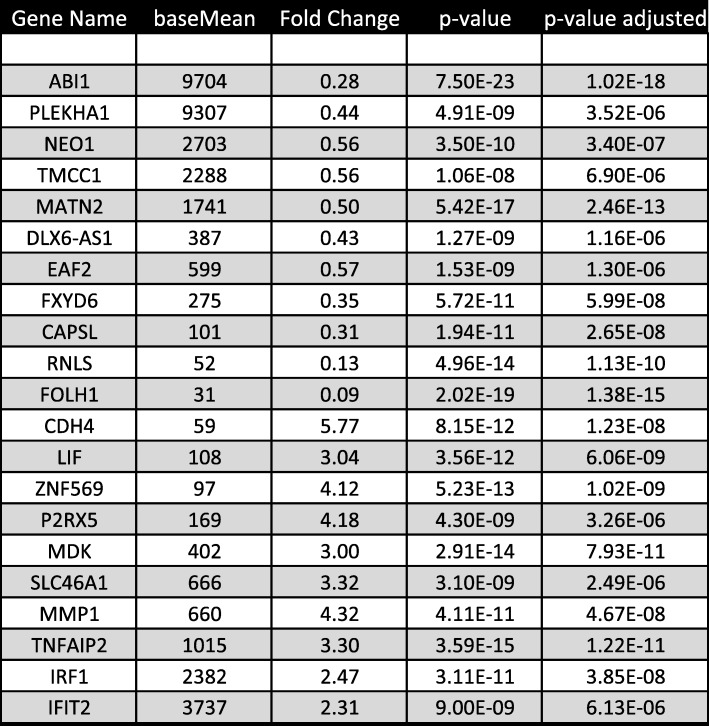
Top 20 Differentially expressed genes

### Loss of ABI1 leads to activation of STAT3 downstream of the non-canonical WNT5a FZD2 receptor

RNA sequencing analysis indicated that ABI1 depletion promoted non-canonical WNT signaling (Fig. 6A). Of particular interest was upregulation of the non-canonical FZD2 receptor, which can be stimulated with WNT5a ligand in metastatic tumor cell lines [20]. Moreover, we observed increased phosphorylation of STAT3 on tyrosine 705 and serine 727 in ABI1 KO cell lines (Fig. 6B), thus indicating transcriptional activation of STAT3 upon its nuclear translocation [41]. We also found that the Src family kinase FYN, which is also implicated in the pathway was upregulated in one of the cell lines (Fig. 6B). Re-expression of ABI1 rescued the activation of STAT3 back to the wild-type level. STAT3 was co-immunoprecipitated with ABI1 and FYN, thus confirming the ABI1-FYN-STAT3 axis (Fig. 6C). Experiments using the recombinant ABI1 isoforms and a different set of FYN antibodies also confirm the complex immunoprecipitation (Additional file 6: Figure S6A-D). Moreover, ABI1 loss led to enhanced nuclear localization of activated pSTAT3 Y705 as indicated by subcellular fractionation (Fig. 6D) and immunofluorescence localization experiments (Fig. 6E-F). Phosphorylated nuclear pSTAT3 Y705 can be observed in PCa tumors with low ABI1 expression (Fig. 6G).

## Discussion

Here, we investigated ABI1 downregulation as a mechanism by which prostate tumors gain invasive and metastatic potential. Examination of tumor tissue indicated low ABI1 expression in in patients with high-grade prostate tumors and an association between low ABI1 and clinically significant events (biochemical recurrence, metastasis, and death). These data support the role of *ABI1* gene inactivation in prostate tumor progression. Although *ABI1* gene loss has previously been associated with highly aggressive metastatic prostate tumors [10], the mechanism by which inactivation of *ABI1* promotes invasion remained unclear.

### Downregulation of ABI1 expression in tumor tissue is associated with tumor progression from low to high-grade tumors

Digital image analysis of ABI1 expression indicated significant downregulation of the protein expression in Gleason 5 pattern in comparison to pattern 3 and 4 tumors. Gleason pattern 5 is characterized by loss of glandular structure, sheets of cells and single cells with mesenchymal-like morphology. This observation is consistent with downregulation of cell-cell adhesion promoting loss of gland morphology due to loss/downregulation of ABI1 and its role in promoting epithelial to mesenchymal transition. Moreover, the apparent upregulation of ABI1 levels in the transition from benign to low grade tumors indicate a potential failsafe mechanism that initially prevents tumor progression, which when broken, leads to aggressive tumors with high metastatic potential.

### Downregulation of ABI1 occurs through mutations, deletions, and aberrant splicing

Mutations of *ABI1* in prostate cancer are not very common but are evident in tumor sequencing data [10, 38] and PCa cell lines. The mutations lead to low ABI1 expression (see Fig. 1b). DU145 cells have a mutation in the SH3 domain of ABI1, M468I (cBioportal, [42]. The LNCaP cell line has an aberrant splice site *ABI1*-exon 6 skipping mutation affecting the interactions with c-ABL and the p85 subunit of PI3 kinase [43, 44]. Moreover, there were striking aberrations in the expression of alternative spliced variants of Abi1 in prostate cancer cell lines and tumor organoids identified here (Fig. 1C-D). The *ABI1* gene has been listed as one of the top splice-isoform dysregulated genes in human cancer [45]. Moreover, *ABI1* is target of splicing factor SRRM4, which is overexpressed in neuroendocrine prostate cancer (NEPC) [46]. Interestingly, NEPC tumors and NEPC cell line models exhibit expression of specific splice variants of ABI1 [46, 47]. Isoform splice aberrations may not be related to simple splicing events or somatic mutation events [45]; hence, the mechanism by which ABI1 downregulation contributes to prostate tumorigenesis may extend beyond the overall protein level downregulation.

Apart from the consequences of loss of Exon 6 discussed above, ABI1 isoforms exhibit functional differences in regulating actin cytoskeleton dynamics through loss of exon 10 mutations [38] and binding to activated RAC1 [34] or ENA/VASP [48], which may contribute to the role of ABI1 in regulating tumorigenic phenotype, as demonstrated here by differential rescue of the ABI1-null phenotype (compare the isoform 2 and 3 rescue effect shown in Fig. 4 and Fig. 5). The mechanism of ABI1 isoform dysregulation is unknown, but the evidence of widespread downregulation of ABI1 in high-grade prostate tumors (PCa-1 and 2 cell lines) warrants further inquiries.

### We propose that ABI1 loss induces an invasive phenotype through two major mechanisms, activation of the EMT program and loss of stability of the WAVE complex, leading to disruption of cell-cell adhesion

The spheroid model demonstrates a critical role of ABI1 in cell-cell adhesion. ABI1 and proteins of the WAVE complex have been found to have key roles in formation and maintenance of proper epithelial cell-cell adhesion [27, 28, 49]. Downregulation of E-cadherin was found in the Abi1 KO mouse prostate, which demonstrated loss of epithelial architecture and hyperproliferation [38]. These findings indicate loss of cell-cell adhesion as a mechanism contributing to tumor suppression due to ABI1 loss.

### Induction of a migratory phenotype upon dysregulation of integrin signaling supports the role of ABI1 in controlling invasion

Significant evidence has demonstrated the critical role of integrins in the prostate cancer invasive phenotype and bone metastasis [50]. Abrogation of β1 integrin signaling decreased metastasis to bone and lymph nodes by PC3 cells [51]. Moreover, αVβ6 promotes MMP2 activity [52]. RWPE-1 ABI1 KO cells demonstrated upregulation of MMP1 in RNA sequencing data and simultaneous upregulation of several integrins, which is consistent with the high migratory activity of the 3D spheroids lacking ABI1.

### Genome-wide expression and pathway analysis of RNA sequencing data points to Abi1 as a key regulator of integrin signaling

Integrin α3, 5, X, β1 and 2 were upregulated in the absence of ABI1 but rescued by re-expression of ABI1. Considering the fact that integrin recycling occurs via rapid recycling of dorsal ruffles and that ABI1 is the critical regulator of dorsal ruffling [29] [34], we propose that loss of ABI1 impedes cytoplasmic recycling of integrins, thus leading to increased integrin levels. Interestingly, β4 integrin is downregulated in ABI1 KO cell lines. The mechanism underlying this observation is unclear, but the Abi1 KO phenotype is consistent with the invasive phenotype of siRNA-mediated β4 integrin disruption in RWPE-1 cells [53]. The global effect on integrin levels in RWPE-1 ABI1 KO cells indicate a critical role of ABI1 in integrin function and thus in regulating the invasive potential of cells. ABI1 might directly interact with integrins, as demonstrated for α4 [33], and/or regulate the actin polymerization input into focal adhesions through the WAVE complex. In the absence of ABI1/WAVE complex activity, other actin regulatory complexes, such as NWASP, have been proposed to regulate Apr2/3-dependent actin polymerization to promote invasion [54], but we did not observe upregulation of NWASP (Additional file 4: Figure S4). Notably, we observed increased FAK phosphorylation, thus supporting activation of integrin signaling downstream of FAK. Activation of FAK also provides potential pathway for STAT3 activation either through direct interaction [55] or through FYN [56, 57]. The latter interaction is controlled by ABI1, thus further supporting the tumor suppressor role of ABI1.

### ABI1 controls the invasive phenotype by suppressing the non-canonical WNT signaling

Gain of mesenchymal EMT markers (Fig. 6A) upon *ABI1* disruption and simultaneous upregulation of the non-canonical WNT signaling signature support the hypothesis that ABI1 negatively regulates the WNT5a-activated pathway by sequestering SRC family kinases. This pathway is activated in invasive tumors [20] and was demonstrated in high-grade prostate cancer [21]. We previously reported ABI1 phoshotyrosine pY421 binding to the SH2 domain of Src family kinases; the FYN-SH2 domain displays particularly high affinity [43]. Moreover, the ABI1 KO RWPE1 cell line expresses FYN and FZD2. Loss of Abi1 leads to STAT3 activation and enhanced nuclear localization, which can be rescued by ABI1 re-expression; ABI1 is found in the complex with FYN and STAT3 (Fig. 6C, Additional file 6: Figure S6), although it is not clear whether ABI1 interacts with STAT3 directly or this occurs through FYN. In summary, we propose that ABI1 acts as a gatekeeper to sequester FYN-mediated activation of the signal between FZD2 receptor and STAT3-dependent activation of EMT transcription (Fig. 6C). The WNT5a-FZD2-STAT3 pathway is activated in high-grade prostate tumors [21], as well as in liver, lung, colon and breast cancer cell lines [20]. Inactivation of *ABI1* in bone marrow also leads to activation of STAT3, hence ABI1 is likely to be universal regulator of the STAT3 pathway [58]. The absence of nuclear localization of β-catenin or phosphorylation changes in RWPE-1 ABI1 KO cells (Additional file 5: Figure S5) and the lack of evidence of a β-catenin driven TCF/LEF transcriptional signature suggests that ABI1 does not play a role in the canonical β-catenin-dependent WNT pathway, at least in epithelial cells.

## Conclusions

We investigated the mechanism for *ABI1* loss in prostate tumor progression. Downregulation of ABI1 is associated with the high-grade of prostate tumors and is evident in metastatic prostate cell lines. Our mechanistic results support the hypothesis that Abi1 acts downstream of the non-canonical WNT receptor FZD2 and upstream of the active FYN-STAT3 axis to control epithelial plasticity through an EMT program. Hence, ABI1 is a gatekeeper of epithelial tumor invasiveness.

## Materials and methods

### Tissue microarrays

A small tissue microarray (TMA) was constructed using prostate cancer tissues from 32 patients who underwent radical prostatectomy from 2000 to 2012 at Kingston Health Services Center (KHSC). Prostatectomy slides were reviewed and regraded by two urologic pathologists (TJ and DMB) according to ISUP 2014 guidelines (GG1, *n* = 15; GG2, *n* = 3; GG ≥ 3, *n* = 13), and cases were distributed on the TMA (see also Additional file 7). Recurrence was defined as two PSA values of ≥0.2 ng/ml after prostatectomy and/or notation of recurrence in the patient’s chart (recurrence, *n* = 12 (37.5%)). Triplicate 0.6-mm cores were harvested from both high- and low-grade cancer areas as well as benign glands from the same case. The study was approved by institutional Ethics Review Boards at Queen’s University. Immunostaining of ABI1 (Cell Signaling Technology cat. #39444) was performed at Histowiz, Inc., Brooklyn NY 11226.

The Vancouver Prostate Centre Pathology Core Predictive Tissue Microarray Series was built from 505 radical prostatectomies obtained from Vancouver General Hospital. The patients were not exposed to any type of therapy before surgery. The patients had more than 5 years of follow-up time as well as complete clinical data. The TMAs were constructed semi-automatedly by punching duplicate 1-mm cores from each specimen using a TMArrayer from Pathology Devices with an attached Leica M50 stereo microscope. The morphology of each core was scored using the currently recommended ISUP Gleason grouping standards i.e. WHO Grade Groups (Epstein et al., Am J Surg Pathol. 2016; 40:244–52). WHO Grade Groups in this TMA series reflect the normal distribution of scores, with Group 1 and 2 being 60%, Group 3 and 4 being 32% and group 5 being 8%. See Additional file 1: Figure S1B, Contigency Table 1 detailing the heterogeneity of ABI1 expression in cores. To address the intra-core heterogeneity ABI1 expression was evaluated by digital imaging for each Gleason pattern. Using the Leica/ Aperio image scope software, the area of interest, in this case, the specific Gleason pattern were selected and annotated. The software runs an algorithm based system to quantify the number of pixels by their intensity and normalised the data by the selected area and creates an automated macro. The automated digital image analysis was performed using Aperio *Positive Pixel Count*. The final core is represented by a number from 0 to 1. One-way ANOVA was used for expression analysis among Gleason patterns 3–5. Linear-by-Linear association exact test (Sytel Studio-9) was used for ABI1 expression correlation with variables (biochemical recurrence, metastasis, and death).

### Immunohistochemistry

Immunohistochemistry was performed on TMA sections with the automated immunohistochemistry staining platform DISCOVERY ULTRA (Ventana Medical Systems, Inc.). Antigen retrieval was conducted with Cell Conditioning 1 (CC1) (Ventana) at 95 °C for 64 min. Slides were incubated with a 1:200 dilution of Abi1 antibody (Cell Signaling Technologies, 39444S) at room temperature for 2 h. For detection, a DISCOVERY ChromoMap DAB Kit, anti-HQ HRP, and anti-rabbit HQ (Ventana) were used. Digital images of stained TMAs were acquired with an SCN400 Slide Scanner (Leica Microsystems). Positively stained cells were analyzed with Aperio ImageScope (Leica Biosystems).

### Cells and culture

#### 2D cultures

MSK-PCa1, MSK-PCa2, and MSK-PCa3 metastatic human prostate cancer cells and normal-26Nb human prostate cells [39] were grown in human organoid media as described in [39] with the following exceptions: recombinant R-spondin 1 (Peprotech, NJ) and noggin (Peprotech) were added to the organoid media at 500 ng/ml and 100 ng/ml, respectively, as described in [59]. RWPE-1 non-tumor human prostate cells (ATCC) were cultured using a keratinocyte serum-free medium kit (Life Technologies, IL) containing 0.05 mg/ml bovine pituitary extract, 5 ng/ml epidermal growth factor, and 5 μg/ml gentamicin (Life Technologies). NIH-3 T3 mouse embryonic fibroblasts (ATCC) and VCaP human prostate cancer (ATCC) cells were grown in DMEM containing 4.5 g/L glucose, 584 mg/L L-glutamine, and 110 mg/L sodium pyruvate (Corning, Tewksbury, MA) and further supplemented with 100 U/ml penicillin/streptomycin (Corning), 1X non-essential amino acids (Corning), 10 mM HEPES (Corning), and either 10% bovine calf serum (HyClone, Logan, UT) for NIH-3 T3 cells or 10% fetal bovine serum (HyClone) for VCaP cells.

#### 3D cultures

Organoid RWPE-1 cell cultures were established in 50–60% (v/v) Matrigel membrane matrix (Corning) on non-tissue culture treated plates. MSK-PCa and normal-26Nb cells were maintained as continuous 3D organoid cultures in 75% (v/v) Matrigel growth factor reduced (GFR) membrane matrix (Corning) on non-tissue culture treated plates and harvested from and reseeded in fresh Matrigel every 12–14 days. Fresh cell-type specific medium was added three times per week for all organoid cultures. Organoids were harvested from the Matrigel plug by adding dispase (Life Technologies) at 1 mg/ml (final volume) to the medium, followed by scraping of the well contents with a cell lifter, trituration, incubation for 2 h at 37 °C in a cell culture incubator, and centrifugation at 250×g for 4 min at 4 °C. MSK-PCa and normal-26Nb organoids were then resuspended in TrypLE Express (Life Technologies) supplemented with 1.8 U/ml dispase and incubated at 37 °C with trituration every 5 min until organoids dissociated into a single cell suspension, which was centrifuged again for downstream application or replating. RWPE-1 organoids were treated with 0.05% Trypsin-EDTA for 5 min at 37 °C, which was then neutralized using 2% FBS in PBS, and the organoid cells were centrifuged.

#### 3D overlay cultures

RWPE-1 cell lines were plated on collagen-coated glass-bottomed chamber slides (Ibidi) and allowed to attach overnight. Then, the medium was removed, and 50% v/v Matrigel was added on the cells, which were cultured for another 3 days and then imaged on a Molecular Devices ImageXpress Micro Confocal High-Content Imaging System. Images were quantified for “Shape factor” using MetaXpress® software.

### Developing ABI1 CRISPR and rescue cell lines

The target sequence identification for designing gRNA was performed using the online software crispr.mit.edu. The gRNA sequence CTAGAGGAGGAGATCCCGTC (TGG) in exon 1 of *ABI1* was cloned into a pCas-Guide-EF1a-GFP vector (cat.#: GE100018) from Origene Technologies (Rockville, MD). RWPE-1 cells were transfected with the CRISPR plasmid using Lipofectamine 3000 transfection reagent (Thermo Fisher Scientific). Then, the cells were trypsinized and sorted via fluorescence-activated cell sorting (FACS) to obtain GFP-positive cells using a Becton Dickinson FACSAria III Cell Sorter. Clonal selection of single GFP-positive cells yielded several surviving clones, which were screened for Abi1 protein levels via western blotting, followed by sequence verification by sequencing with Abi1 Exon1 specific primers as described [38]. ABI1 Isoform 2 and Isoform 3 constructs were cloned into a pMSCVpuro backbone (Addgene) and packaged into retrovirus using Phoenix-AMPHO producer cells (ATCC). RWPE-1 ABI1 KO clone 35 cells were transduced for two rounds with the retrovirus produced, and the resistant pool was selected with puromycin for use as “rescue” cell lines.

### Western blot analysis

Whole-cell extracts were prepared by lysing cells in 50 mM Tris-Cl pH 7.5, 10 mM MgCl_2_, 0.5 M NaCl, 2% (v/v) NP-40, 0.01% (v/v) benzonase (Sigma-Aldrich, St. Louis, MO), 1% (v/v) protease inhibitor cocktail (Sigma-Aldrich), and 1X phosphatase inhibitor cocktail set V (Millipore Sigma, Billerica, MA). Total protein concentrations were determined using Precision Red Advanced (Cytoskeleton, Denver, CO) or Pierce 660 nm (Thermo Scientific) protein assays. Electrophoresis and blotting of protein extracts were performed using mini-PROTEAN tetra handcast and trans-blot turbo transfer systems (Bio-Rad, Hercules, CA). The primary antibodies used were mouse anti-Abi1 (MBL International, clone 1B9), goat anti-Abi2 (Santa Cruz, P-20), rabbit anti-Abi3 (Genetex, GTX60228), mouse anti-WAVE1 (Millipore, clone K91/36), rabbit anti-WAVE2 (Santa Cruz, H-110), rabbit anti-WAVE3 (Cell Signaling, 2806), rabbit anti-Brk1 (LifeSpan BioSciences, LS-C82659), rabbit anti-GAPDH (Sigma G9545), mouse anti-β-actin (Sigma-Aldrich, clone AC-15), rabbit anti-Cyfip1 (ThermoFisher PA5–31984), rabbit-anti-Nap1 (ThermoFisher PA5–30406), rabbit anti-E-cadherin (Santa Cruz 7870), rabbit anti-β-catenin (Santa Cruz 7199), mouse anti-Integrin α3 (Santa Cruz 374,242), mouse anti-Integrin α5 (Santa Cruz 166,665), mouse anti-Integrin αX (Santa Cruz 46,676), rabbit anti-Integrin β1 (Cell Signaling 4706), rabbit anti-Integrin β2 (Cell Signaling 73,663), mouse anti-Cytokeratin8 (Santa Cruz 8020), mouse anti-Fibronectin (Santa Cruz 8422), mouse anti-SLUG (Santa Cruz 166,476), mouse anti-HNF-3β (Santa Cruz 101,060), rabbit anti-Fyn (Cell Signaling 4023), goat anti-FYN (BioRad VPA00019), mab anti-FYN (BioRad VMA00053), rabbit anti-PARP (Cell Signaling 46D11), rabbit anti-Stat3 (Cell Signaling 4904), rabbit anti-p-Stat3 Y705 (Cell Signaling 9145), rabbit anti-p-Stat3 Y727 (Cell Signaling 9134), rabbit anti-ZO-1 (Thermo Scientific 40–2200), rabbit anti-p-β-catenin S675 (Cell Signaling 9567), rabbit anti-p-β-catenin T41/S45 (Cell Signaling 9565), rabbit anti-p-β-catenin S552 (Cell Signaling 9566), and rabbit anti-N-WASP (Cell Signaling 4848). The secondary antibodies (Thermo Scientific) were donkey anti-goat, goat anti-mouse, or goat anti-rabbit IgG HRP conjugates. Blots were imaged using supersignal west pico, pico-plus, or femto chemiluminescent substrate (Thermo Scientific) on a PXi touch imaging system, and signal was quantified using GeneTools software (Syngene, Frederick, MD). The data were analyzed using one-way ANOVA and Student’s t-test.

### Immunoprecipitation

Cells were EGF/BPE-starved (using KSFM-only medium) overnight, followed by EGF stimulation for 10 min along with 10 mM pervanadate treatment (10 mM sodium orthovanadate + 0.3% hydrogen peroxide in KSFM). Cells were immediately washed with cold PBS and lysed using Pierce IP lysis buffer with protease and phosphatase inhibitors. The resulting lysate was immunoprecipitated using anti-Abi1 antibody (clone 1B9, MBL) at an antibody:lysate ratio of 1:100. Immunoprecipitation was performed using Dynabeads (ThermoFisher) according to the manufacturer’s instructions, except the incubation was performed overnight at 4 °C and preceded by a pre-clearing of the lysate with IgG under the same conditions.

### Live cell imaging

RWPE-1 cell lines (parental, control clone 16, ABI1 KO clone 12, ABI1 KO clone 35, Rescue Isoform 2 and Rescue Isoform 3) and PCa cell lines (MSK-PCa1, MSK-PCa2, and MSK-PCa3) were plated in regular or GFR Matrigel, respectively, on non-tissue culture treated plates. Freshly defrosted cells were used for PCa cell lines, and early passage cells were used for RWPE-1 cell lines. The former were imaged live overnight 4 days post-culture, and the latter were imaged at 7 days. Images were taken with a Molecular Devices ImageXpress Micro Confocal High-Content Imaging System, and images were merged from 3 independent z-planes once every 5 min for 14 h with a 20X extra-long working distance objective while cells were maintained at 37 °C and 5% CO_2_. Images were analyzed, and movies were prepared using MetaXpress® and ImageJ software. The data were analyzed using one-way ANOVA and Student’s t-test.

### Immunostaining

RWPE-1 cells were plated on 12-mm round #1.5 glass coverslips (Thomas Scientific, NJ). At ~ 50% confluence, they were rinsed with PBS, fixed with 4% paraformaldehyde (PFA) for 10 mins at room temperature, and washed with PBS 3 times, before being blocked with PBSAT (PBS, 1% BSA, 0.5% Triton X-100) for 30 min at room temperature. Coverslips were then incubated with primary antibody in PBSAT overnight at 4 °C, washed with PBSAT 3 times, and incubated with secondary antibody for 1 h at room temperature, followed by 3 more washes with PBSAT. Finally, the coverslips were rinsed with dH_2_O and mounted on glass slides using Prolong Diamond antifade mountant with DAPI (Thermo Fisher Scientific) and imaged with the Molecular Devices ImageXpress Micro Confocal High-Content Imaging System. Images were processed using MetaXpress® and ImageJ software.

### Scratch wound assay

RWPE-1 cells (controls, Abi1 KO, and rescue) were plated in 12-well plates and grown to 100% confluence. Then, scratches were made in the confluent monolayer using a 200-μl pipet tip. The plates were imaged every day for 4 days using a 4X objective to view the cells migrating into the scratch wound. The covered area was quantified using ImageJ, and migration was analyzed using paired Student’s t-tests.

### Proliferation assay

Cells were plated in triplicate in a 96-well plate and measured at 2, 3, and 4 days using a CyQUANT NF Cell Proliferation Assay Kit (Invitrogen) according to the manufacturer’s instructions. The data were normalized to the day 2 data and analyzed using two-way ANOVA with multiple comparisons.

### RNA sequencing and analysis

RNA was extracted from cell pellets using an RNeasy Mini Kit (Qiagen). RNA quality and quantity were assessed with an Agilent 2100 Bioanalyzer using an RNA 6000 Nano Kit. For each sample, 2 μg of total RNA was processed using an Illumina TruSeq Stranded mRNA Library Prep Kit following the manufacturer’s protocol. The sizes of the sequencing libraries were evaluated with the Agilent 2100 Bioanalyzer using a DNA 1000 Kit. Each library was then quantified using a Qubit 3.0 fluorometer (ThermoFisher). Individual libraries were diluted to 4 nM and then pooled together for sequencing. The library pool was sequenced on an Illumina NextSeq 500 instrument using a NextSeq 500/550 High Output Kit, with a loading concentration of 0.8 pM and a paired end 2 × 75 bp read. A 1% spike-in of PhiX control library (Illumina) was included in the run.

### RNA-seq data processing and gene expression analysis

We analyzed gene expression profiles where Abi1 KO clones 12, 4, and 35 were compared to controls: clone 15, 16, and RWPE-1. For RNA-seq alignment, the read length was 75 nt and primary number of reads were between 56.2 10^6^ and 76.8 10^6^. Alignment information: BAM format files; percentage of total aligned were between 95.9 and 98.78%; reads with spliced alignment were between 24.20 and 25.60%. Exonic read counts in the samples ranged between 47.0 10^6^ and 60.7 10^6^. Differentially expressed genes (DEGs) were compared between control and test samples. Differential gene expression (DEG) was determined using RNA express v.1.1.1.0, and gene annotation was performed using HG19 (GENCODE) via The BaseSpace Sequence Hub User Interface. We identified 441 DEGs at q < 0.05 and log2 (fold change) ≥1. Gene enrichment (GO) analysis and Reactome Pathway Enrichment Analysis (via Panther DB http://www.pantherdb.org/) was used for identification of overrepresented gens and pathways.

### Subcellular fractionation

To isolate subcellular fractionations we used NE-PER reagents from Thermo Fisher (Cat. No.78833). PARP and GAPDH antibodies were used as markers of the nuclear and cytoplasmic fraction, respectively.

### Statistical analysis

All cell line data were analyzed using GraphPad Prism software. One-way, two-way ANOVA and Student’s t-test were used to determine significance; *p*-value < 0.05. Categorical data and multivariate analyses were carried out using Sytel Studio-9 software (Sytel Inc. Pume).

## Additional files

Additional file 1: **Figure S1.** Evaluation of ABI1 expression in a test TMA. A) ABI1 expression is downregulated in prostate tumors with high Gleason grade pathology. The ABI1 expression levels were compared with the disease stage of the tumors. For KHSC cohort (n=32), binary scoring method was used as described in materials and methods section which categorizes patients into two (down/up) distinct groups based on arbitrarily defined threshold of % positive cells (>20%) and overall staining intensity as compared to internal positive controls (benign glands). Intra- and inter-core heterogeneity (i.e. mosaic pattern) were defined when cancer cells with decreased or retained ABI1 protein were observed within the same (intra) or different (inter) TMA cores and it was assessed manually. Subsequently, the case was considered homogeneous, when there was no inter-or intra-core heterogeneity seen. In this small TMA cohort within the cases with ABI1 down-regulation, we have observed 4 cases with inter-core heterogeneity, 2 cases with intra-core heterogeneity and one case with IDC with heterogeneous/mosaic pattern. (B) ABI1 expression and prostate tumor heterogeneity. Left, Contingency Table for ABI1 expression levels in Gleason patterns associated with different WHO grade groups. Gleason 3-3, Grade group 1; Gleason 3-4, Grade group 2; Gleason 3-3, Grade group 3; Gleason 4-4 and Gleason 5-3, Grade group 4; Gleason 4-5 and Gleason 5-5, Grade group 5. ABI1 expression was quantified by digital imaging as described in Material and methods and presented here as group of staining intensity: 0, negative staining, 1, weak staining; 2, moderate; 3, strong (digital scoring). Digital scoring takes into consideration % of cells stained for each level of intensity in a tumor core and is highly quantitative. The normalized stain intensity represents average intensity per core calculated as follow takes into consideration percent of cells stained for each intensity level (0, 1, 2 and 3), the average stain intensity for each core is calculated as sum of intensities and ranged from 0-1. The digital scoring showed statistically significant correlation with manual H-score as determined by direct comparison of digital vs. manual scoring of 505 patients in the TMA.The table below lists ABI1 expression level in tumors associated with each WHO tumor Grade Group histopathology, Group 1-5, and in benign tissue. On the right an image demonstrating intra-core tumor heterogeneity of ABI1 expression with Benign, and Gleason 4 and 5 patterns; Benign, represents normal prostate tissue (VPC cohort). See Figure 1B for quantification of ABI1 expression. (JPG 2994 kb)

Additional file 2: **Figure S2.** Generation of Abi1 CRISPR KO in RWPE-1 cells. (A) ABI1 exon 1 sequence, with guide RNA sequence marked in red and PAM marked in blue. (B-F) Western blots showing ABI1 and WAVE2 for screening of CRISPR clones; WAVE2 downregulation is correlated with levels of Abi1. β-Actin was used as the loading control. (G) Sequencing analysis of selected clones. Black text shows the wild-type sequence, green shows wild-type clones, blue shows heterozygous KO clones, and red shows homozygous KO clones. (JPG 1147 kb)

Additional file 3: **Figure S3.** Loss of Abi1 causes downregulation of the WAVE complex and cell-cell adhesion markers in mouse embryonic fibroblasts. (A) Representative western blots showing reductions in WAVE1 and WAVE2 in Abi1 KO MEFs. (B) Western blots showing reductions in the adherens junction proteins N-cadherin and β-catenin and a modest decrease in the tight junction marker ZO-1 in Abi1 KO MEFs. β-Actin was used as the loading control. (C) Immunostaining for N-cadherin and β-catenin showing loss of cell-cell junctional staining of the proteins in the Abi1-null MEFs (bottom panel) compared with the control cells (top panel). Scale bar represents 10 µm. (JPG 579 kb)

Additional file 4: **Figure S4.** *ABI1* KO RWPE-1 cells exhibit no significant increase in proliferation but an upregulation of p-Akt. (A) Proliferation assays were performed at 2, 3 and 4 days post-plating in control RWPE-1 (parental and clone 16), ABI1 KO (clone 12 and 35), and ABI1 rescue (Iso2 and Iso3) cells, showing no significant difference in proliferation rates (1-way ANOVA). (B) Western blot showing that p-Akt is increased in ABI1-null cells and can be stimulated by EGF after starvation. β-Actin was used as the loading control. (JPG 595 kb)

Additional file 5: **Figure S5.** Generated *ABI1* KO cells exhibit no change in p-β-catenin or N-WASP. (A) Representative western blots showing no change in p-β-catenin, total β-catenin or N-WASP upon ABI1 loss. Blot shows parental RWPE-1 cells, two control clones and three ABI1 KO clones. β-Actin was used as the loading control. (JPG 461 kb)

Additional file 6: **Figure S6.** FYN and STAT3 interact with the recombinant ABI1 in RWPE cells. (A) Confirmation of ABI1-FYN-STAT3 complex using FYN antibody VPA00019 (BioRad). ABI1 was immunoprecipitated with MBL Mab 1B9: For Western blotting we used Abi1 CST 39444 (bottom panel), STAT3 CST 30835 (middle panel); FYN immunoreactivity was detected with BioRad VPA00019. (B) Recombinant ABI1 interacts with STAT3 (middle panels), and co-immunoprecipitated active phospho-SRC activity as indicated by pan phospho-SRC family kinase antibody to pY416 (CST 2101), lower panels. Left side depicts ABI1 isoform 2 immunoprecipitation; Right side depicts ABI1 isoform 3 immunoprecipitation. (C, D) FYN was co-immunoprecipated with either the recombinant ABI1 isoform 2 (C) or ABI1 Isoform 3 (D) using ABI1 MBL 1B9 antibody. FYN immunoreactivity was detected with BioRad VPA00019; Abi1 with CST 39444; Input, RWPE-1 cell lysate; FT, flow-through, unbound fraction of the lysate; IgG, control immunoprecipitation using isotype IgG as control but lacking ABI1 antibody; IP, immunoprecipitation including the anti-ABI1 antibody. Asterisk in panels indicate the corresponding bands in input/ flow-through and IP fractions. (TIF 1470 kb)

Additional file 7: Excel file listing 441 differentially expressed genes (DEGs) of Abi1 KO RWPE cell lines. (XLSX 160 kb)

Additional file 8: **Movie S1(a-f).** Time-lapse movies of Abi1 KO cell lines (14hrs). Movie S1a: RWPE-1 parental; Movie S1b: Control clone #16; Movie S1c: Abi1 knock-out clone; Movie S1d: Abi1 knock-out clone #35; Movie S1e: Abi1 rescue of Isoform 2; Movie S1f: Abi1 rescue of Isoform 3. (ZIP 11074 kb)
