# Supplementary figures and images for: Abi1 loss drives prostate tumorigenesis through activation of EMT and non-canonical WNT signaling
Source: Cell Commun Signal. 2019 Sep 18;17:120. doi: 10.1186/s12964-019-0410-y (PMC6749699; doi:10.1186/s12964-019-0410-y)

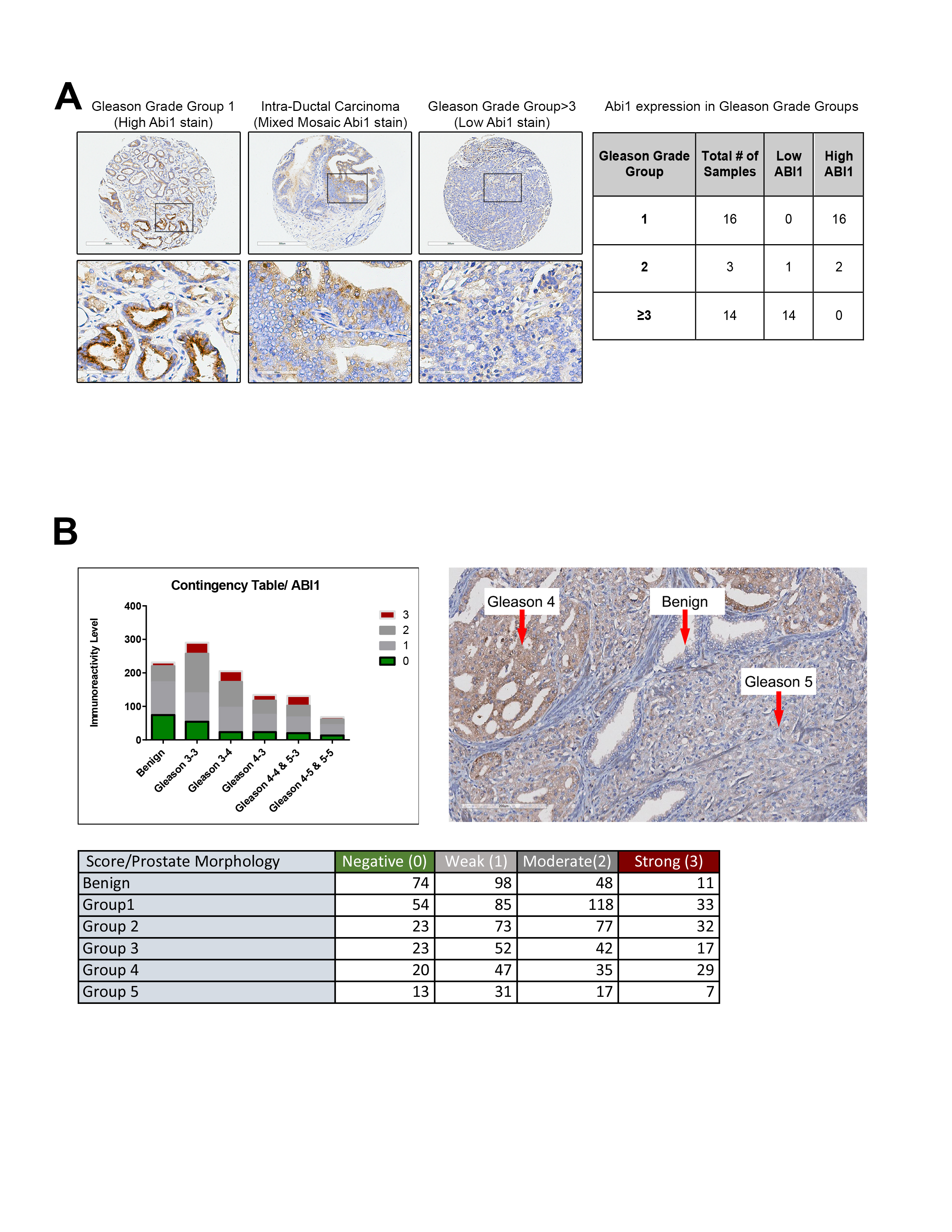

Supplement: Supplementary file 1 — Figure S1. Evaluation of ABI1 expression in a test TMA. A) ABI1 expression is downregulated in prostate tumors with high Gleason grade pathology. The ABI1 expression levels were compared with the disease stage of the tumors. For KHSC cohort (n=32), binary scoring method was used as described in materials and methods section which categorizes patients into two (down/up) distinct groups based on arbitrarily defined threshold of % positive cells (>20%) and overall staining intensity as compared to internal positive controls (benign glands). Intra- and inter-core heterogeneity (i.e. mosaic pattern) were defined when cancer cells with decreased or retained ABI1 protein were observed within the same (intra) or different (inter) TMA cores and it was assessed manually. Subsequently, the case was considered homogeneous, when there was no inter-or intra-core heterogeneity seen. In this small TMA cohort within the cases with ABI1 down-regulation, we have observed 4 cases with inter-core heterogeneity, 2 cases with intra-core heterogeneity and one case with IDC with heterogeneous/mosaic pattern. (B) ABI1 expression and prostate tumor heterogeneity. Left, Contingency Table for ABI1 expression levels in Gleason patterns associated with different WHO grade groups. Gleason 3-3, Grade group 1; Gleason 3-4, Grade group 2; Gleason 3-3, Grade group 3; Gleason 4-4 and Gleason 5-3, Grade group 4; Gleason 4-5 and Gleason 5-5, Grade group 5. ABI1 expression was quantified by digital imaging as described in Material and methods and presented here as group of staining intensity: 0, negative staining, 1, weak staining; 2, moderate; 3, strong (digital scoring). Digital scoring takes into consideration % of cells stained for each level of intensity in a tumor core and is highly quantitative. The normalized stain intensity represents average intensity per core calculated as follow takes into consideration percent of cells stained for each intensity level (0, 1, 2 and 3), the average stain intens [file 12964_2019_410_MOESM1_ESM.jpg]

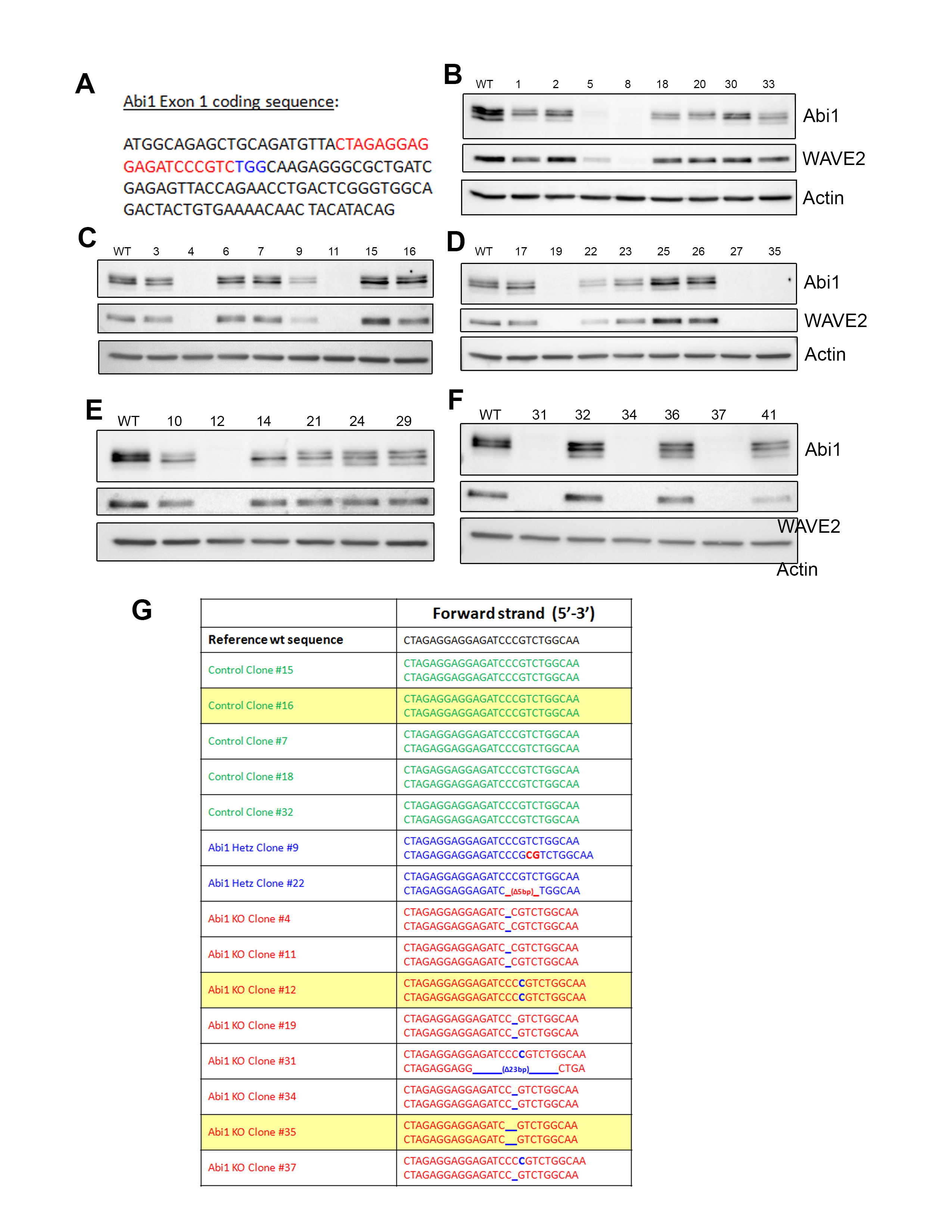

Supplement: Supplementary file 2 — Figure S2. Generation of Abi1 CRISPR KO in RWPE-1 cells. (A) ABI1 exon 1 sequence, with guide RNA sequence marked in red and PAM marked in blue. (B-F) Western blots showing ABI1 and WAVE2 for screening of CRISPR clones; WAVE2 downregulation is correlated with levels of Abi1. β-Actin was used as the loading control. (G) Sequencing analysis of selected clones. Black text shows the wild-type sequence, green shows wild-type clones, blue shows heterozygous KO clones, and red shows homozygous KO clones. (JPG 1147 kb) [file 12964_2019_410_MOESM2_ESM.jpg]

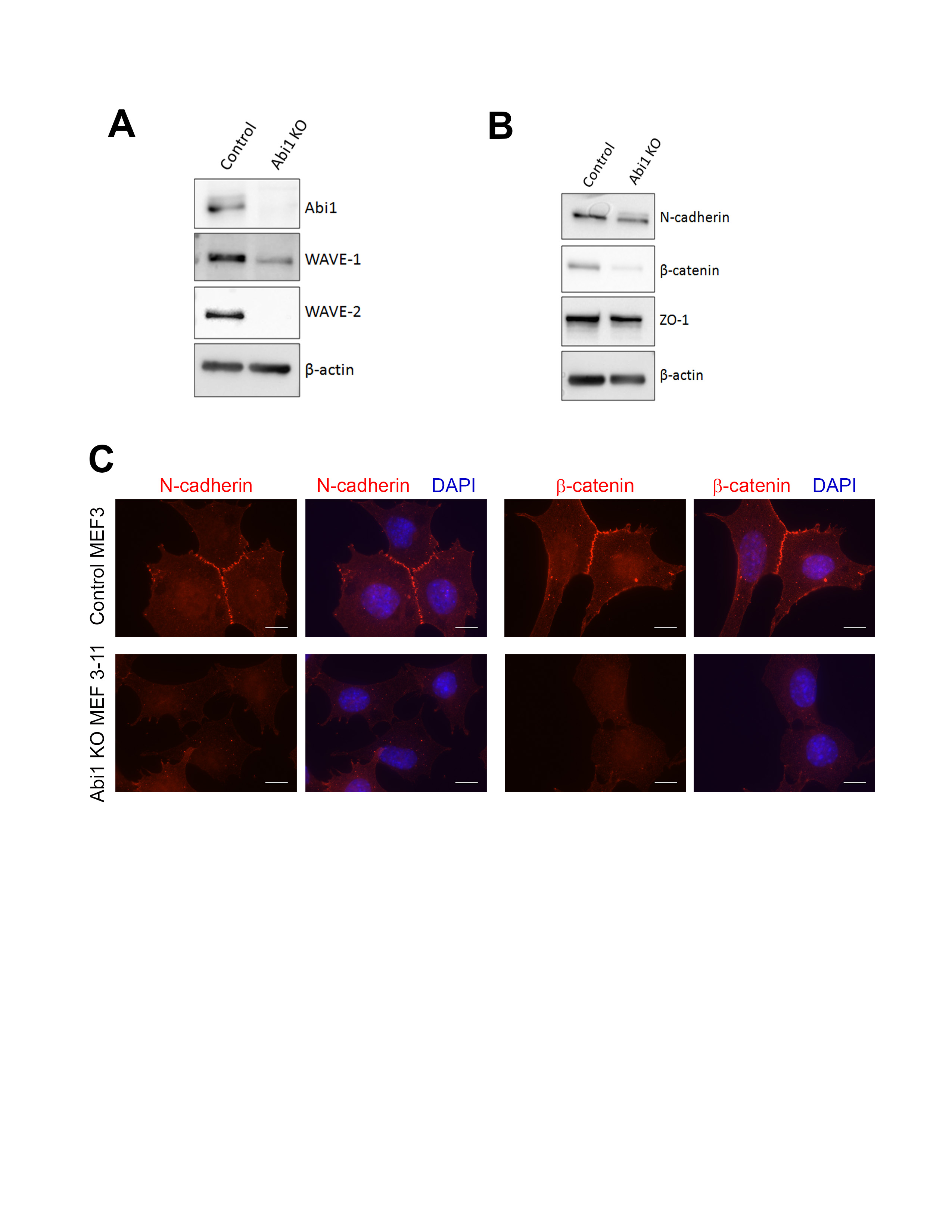

Supplement: Supplementary file 3 — Figure S3. Loss of Abi1 causes downregulation of the WAVE complex and cell-cell adhesion markers in mouse embryonic fibroblasts. (A) Representative western blots showing reductions in WAVE1 and WAVE2 in Abi1 KO MEFs. (B) Western blots showing reductions in the adherens junction proteins N-cadherin and β-catenin and a modest decrease in the tight junction marker ZO-1 in Abi1 KO MEFs. β-Actin was used as the loading control. (C) Immunostaining for N-cadherin and β-catenin showing loss of cell-cell junctional staining of the proteins in the Abi1-null MEFs (bottom panel) compared with the control cells (top panel). Scale bar represents 10 µm. (JPG 579 kb) [file 12964_2019_410_MOESM3_ESM.jpg]

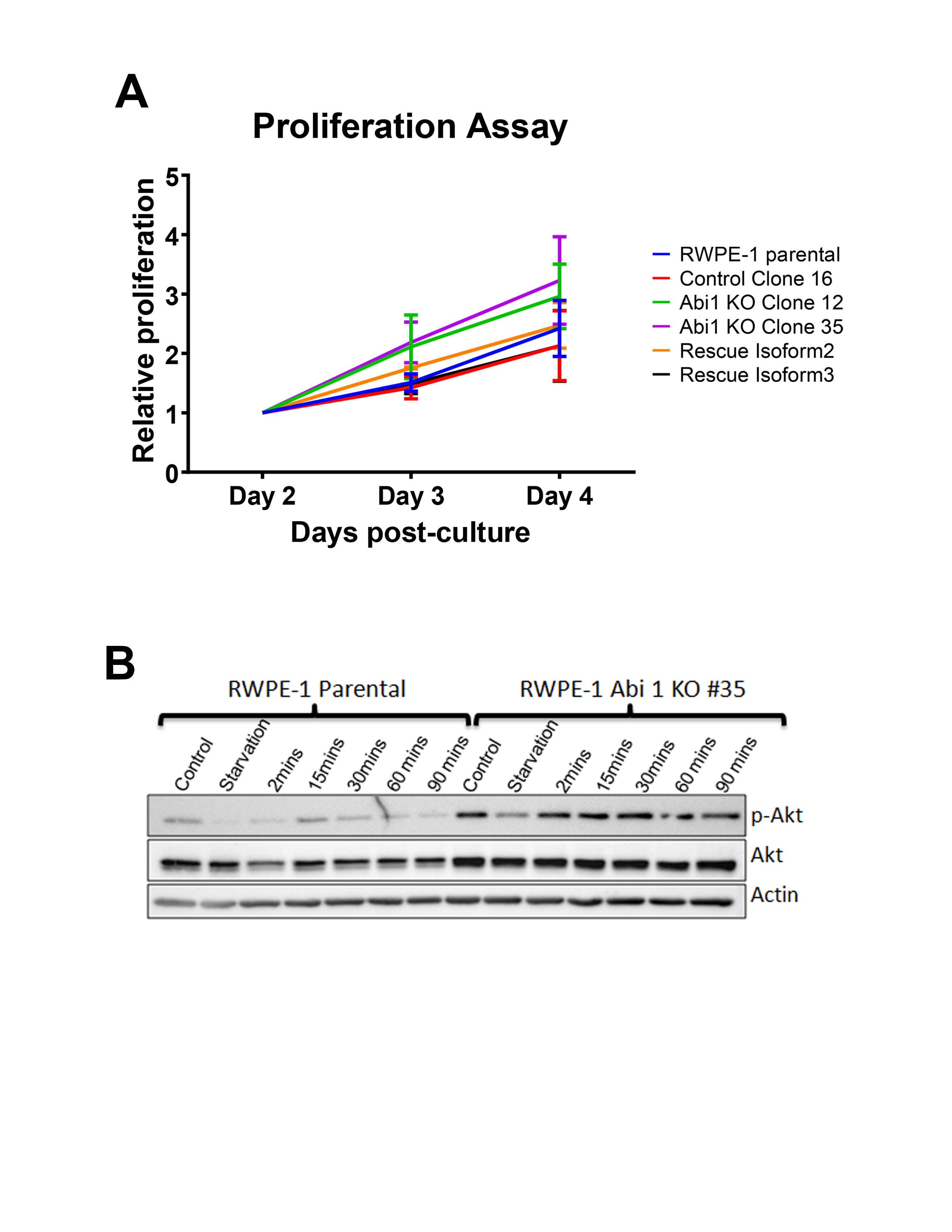

Supplement: Supplementary file 4 — Figure S4. ABI1 KO RWPE-1 cells exhibit no significant increase in proliferation but an upregulation of p-Akt. (A) Proliferation assays were performed at 2, 3 and 4 days post-plating in control RWPE-1 (parental and clone 16), ABI1 KO (clone 12 and 35), and ABI1 rescue (Iso2 and Iso3) cells, showing no significant difference in proliferation rates (1-way ANOVA). (B) Western blot showing that p-Akt is increased in ABI1-null cells and can be stimulated by EGF after starvation. β-Actin was used as the loading control. (JPG 595 kb) [file 12964_2019_410_MOESM4_ESM.jpg]

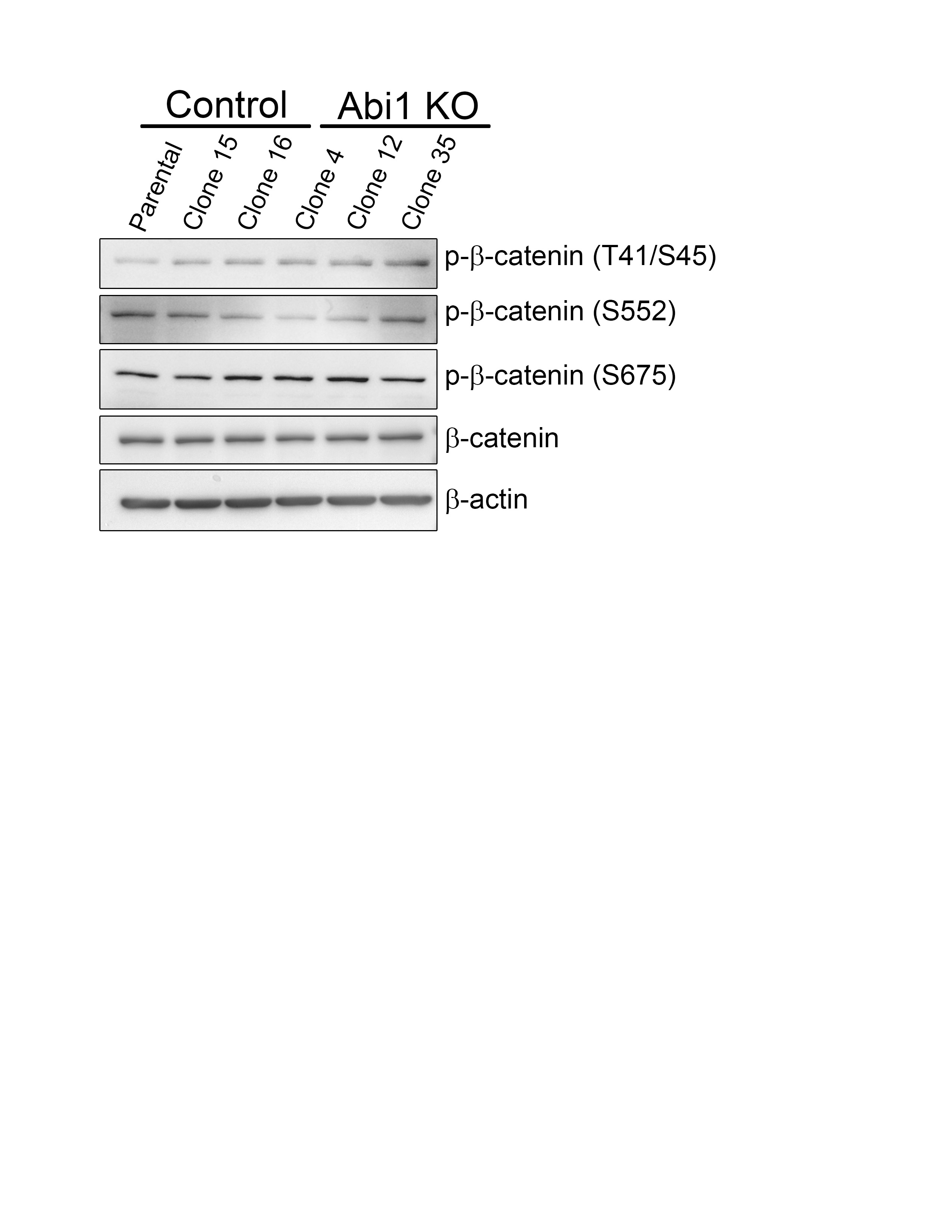

Supplement: Supplementary file 5 — Figure S5. Generated ABI1 KO cells exhibit no change in p-β-catenin or N-WASP. (A) Representative western blots showing no change in p-β-catenin, total β-catenin or N-WASP upon ABI1 loss. Blot shows parental RWPE-1 cells, two control clones and three ABI1 KO clones. β-Actin was used as the loading control. (JPG 461 kb) [file 12964_2019_410_MOESM5_ESM.jpg]
